# Mitigating increasing wildfire risk through fuel break innovations

**DOI:** 10.1016/j.isci.2025.114391

**Published:** 2025-12-10

**Authors:** Nicholas T. Link, Jill F. Johnstone, Xanthe J. Walker, Felecia Amundsen, Hazel K. Berrios, Luc Bibeau, Dorothy Cooley, Ann C. Erickson, Carla Johnston, Joseph M. Little, Nathan Lojewski, Alison D. Perrin, Carly A. Phillips, Stefano Potter, Daniel C. Rees, Lisa B. Saperstein, Jennifer I. Schmidt, Emily E. Sousa, Katie V. Spellman, Andrew Spring, Michelle C. Mack

**Affiliations:** 1 Center for Ecosystem Science and Society and Department of Biological Sciences, Northern Arizona University, Flagstaff, AZ 86011, USA; 2 Institute of Arctic Biology, University of Alaska Fairbanks, Fairbanks, AK 99775, USA; 3 YukonU Research Centre, Yukon University, Whitehorse, YT Y1A 5K4, Canada; 4 Fairbanks Soil and Water Conservation District, Fairbanks, AK 99709, USA; 5 Wildland Fire Management, Government of Yukon, Whitehorse, YT Y1A 2C6, Canada; 6 Teslin Tlingit Council, Lands and Resources, Box 133, Teslin, YT T0A 1B0, Canada; 7 U.S. Department of Interior, Bureau of Land Management, Alaska State Office, 222 W. 7^th^ Ave, #13, Anchorage, AK 99513, USA; 8 Balsillie School of International Affairs, Wilfrid Laurier University, Waterloo, ON N2L3C5, Canada; 9 W. A. Franke College of Business, Department of Accounting, Economics, and Finance, Northern Arizona University, Flagstaff, AZ 86011, USA; 10 Chugachmiut, 1840 Bragaw Street Suite 110, Anchorage, AK 99508, USA; 11 School of Social Sciences and Humanities, Yukon University, Whitehorse, Yukon Y1A 5K4, Canada; 12 Union of Concerned Scientists, 2 Brattle Square, Cambridge, MA 02138, USA; 13 Woods Hole Research Center, Falmouth, MA 02543, USA; 14 U.S. Army Garrison Alaska, Fort Wainwright, Fairbanks, AK 99703, USA; 15 U.S. Fish and Wildlife Service, Anchorage, AK 99503, USA; 16 Institute of Social and Economic Research, University of Alaska Anchorage, 3211 Providence Dr, Anchorage, AK 99508, USA; 17 International Arctic Research Center, University of Alaska Fairbanks, Fairbanks, AK 99775, USA; 18 Geography and Environmental Studies, Wilfrid Laurier University, Waterloo, ON N2L3C5, Canada

**Keywords:** Earth sciences, Climatology, Forestry

## Abstract

A warming climate and expanding wildland urban interface are escalating wildfire risk to human life and property in the boreal forests of western North America. To address this heightened risk, fuel breaks, which reduce fuels and enhance tactical use by firefighters, are increasingly being installed around northern communities. However, the current design and implementation of fuel breaks have social and ecological trade-offs that undermine wider acceptance and adoption. Creative fuel break designs could address these trade-offs by supporting complementary activities with ecological and socio-economic values—termed co-benefits—while maintaining tactical use for wildfire operations. Here, we report results from public listening sessions that recorded desired co-benefits from boreal residents. Through collaboration among scientists, land managers, and local communities, we developed four operationally plausible, innovative fuel break scenarios that provide these co-benefits. Fuel breaks with co-benefits can provide multiple needed services to communities across the region, helping them adapt to a rapidly changing climate.

## Graphical abstract

**Figure:**
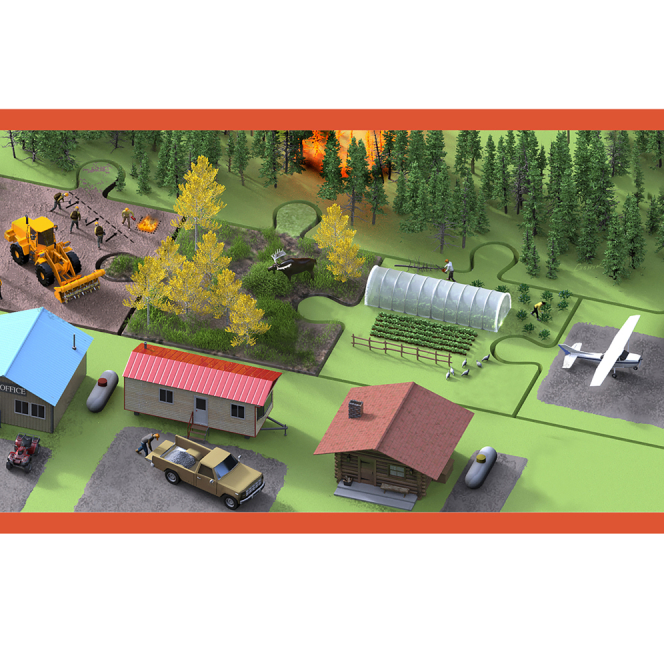

## Introduction

In the boreal forests of western North America, wildfires are an integral part of the landscape that humans have coexisted with over the past 6,000 years.^1^^,^^2^ As climate warms, however, fire seasons are becoming longer and more destructive.^3^^,^^4^^,^^5^ We have exacerbated this problem across the region by rapidly expanding the built environment into undeveloped lands, an area known as the Wildland-Urban Interface^6^ (WUI), and by suppressing natural fires and cultural burning around communities,^1^^,^^7^ increasing the probability that wildfires will threaten society. Combining those factors with budget and staffing limits means that wildland firefighters urgently need a multitude of proactive, tactical tools to protect people and property and to help us return to a safe coexistence with wildfires.^8^

Proactive mitigation of wildfire risk in this ecosystem will require adaptation measures: management and policy actions designed to reduce vulnerability to climate change and its impact on natural disasters.^9^ An emerging adaptation measure in boreal forests for increased wildfire risk is installing fuel breaks. Here we define fuel breaks as patches on the landscape that mitigate risk to communities or other values where land managers remove or restructure flammable vegetation to alter future fire behavior,^10^^,^^11^ compartmentalize the landscape to contain wildfires, and increase firefighter access for active fire line management.^12^^,^^13^ Fuel breaks, along with other adaptation measures at the scale of the homeowner are necessary to reduce wildfire risks to a community. While broader fuel reduction efforts are widespread across the contiguous US and southern Canada,^11^^,^^14^ fuel breaks have only been widely adopted in northern boreal forests over the last twenty years.^15^

Although principally enacted to abate future risk, all adaptation measures come with trade-offs.^16^ For example, maintaining fuel breaks in a low flammability state requires reoccurring treatment and costs^17^ and may compromise ecosystem services such as flood and erosion control,^18^ spiritual and cultural connections to the land,^19^ and habitat space for plants and animals.^20^ Trade-offs can create conflicts between land managers and communities, potentially undermining acceptance or implementation.^21^^,^^22^ Additionally, top-down land management planning that is not place-based may be particularly ill suited in this region because it is home to a large proportion of rural and Indigenous communities that have subsistence lifestyles and strong cultural connections to the lands.^23^ With the relatively recent advent of fuel break use in northern boreal forests, there has been limited research on their social and ecological impacts^24^ or on best ways to mitigate trade-offs.

An approach to reduce trade-offs and conflicts is to integrate co-benefits into the design of adaptation measures.^16^ Co-benefits are secondary to the primary benefit of risk reduction and add additional ecological or socio-economic values at local to global scales.^16^^,^^25^ Redesigning adaptation measures so they provide co-benefits can make them more readily accepted by relevant parties, from local communities to federal agencies.^26^^,^^27^ For example, fuel breaks could be designed as spaces where community members learn about fire science from land managers to help create and sustain fire-adapted communities.^28^ Land cover change associated with fuel removal could create spaces for food production, infrastructure, or recreation sites, thereby generating social and economic opportunities. Fuel break design could allow for the reintroduction of cultural burning to benefit animals and plants that are significant to Indigenous peoples^1^ thereby reducing cultural trade-offs. Further, ecological interventions could create self-sustaining, low-flammability forests that live for decades without high maintenance costs or long-term ecological trade-offs.

The nascent nature of fuel breaks in this region presents a unique opportunity for communities and land management agencies to envision novel adaptation measures for people living in the boreal forest. As the region is tested by a rapidly changing fire regime, tailoring fuel breaks to provide place-based co-benefits can create win-win scenarios that mitigate fire risk while improving health and well-being. Here we report four broad scenarios of fuel breaks with co-benefits developed by an interdisciplinary, international working group of ecologists, social scientists, economists, land managers, and Indigenous representatives. Our scenario development was informed by public listening sessions where 57 members of 31 communities from Alaska, Yukon and the contiguous United States shared their visions of the co-benefits fuel breaks could provide. We coalesced those visions into four plausible scenarios to contribute to ongoing discussions of creative solutions for climate change adaptation at the community and regional level.

## Fire and us: A brief overview of wildfire management in the region

### Fire management over time

Fire has been the dominant large-scale disturbance in the boreal forests of western North America for the last 6,000 years.^29^ Indigenous peoples of the boreal region have had diverse relationships with fire, honing their knowledge systems over millennia of coexistence.^1^ Some peoples actively applied fire on the landscape^30^ and others migrated seasonally to either avoid wildfires or make use of burned areas.^31^ The establishment of permanent settlements during Euro-American colonization dramatically shifted their long-standing relationships with fire through state-enforced fire suppression.^32^ Colonial governments adopted an aggressive approach to wildfire suppression during the 20^th^ century to protect their newly built infrastructure.^23^^,^^33^ Over the next hundred years this stance was softened as scientists and land managers began recognizing the pivotal role of natural disturbances in ecosystem functioning.^23^^,^^33^

**Figure 1:**
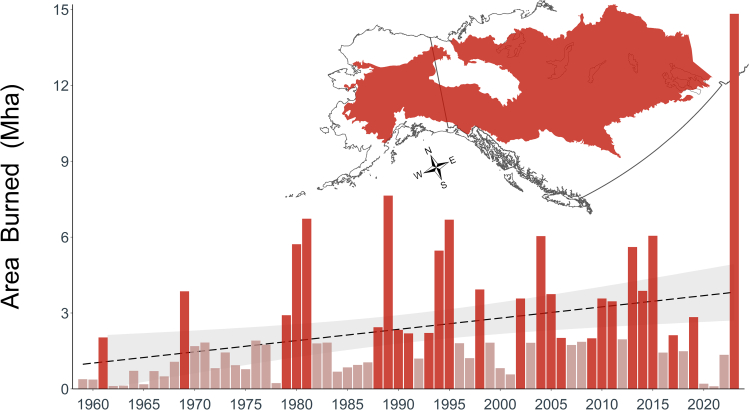
Burned area across the boreal forests of western North America in *Fire History in the Region* Annual area burned in the boreal forests of western North America (inset map). Area burned is increasing, on average, at a rate of over 44,000 ha/year (dashed line shows the trend over time with shading to indicate the 95% confidence interval). Large fire years (>2 Million hectares (Mha), which is larger than the state of New Jersey, USA) noted by the darker shade of red. Since 2000, large fire years are occurring 56.5% of the time (a 78.2% increase compared to the pre-2000 period).

Entering the 21^st^ century, the combination of warmer temperatures,^34^ significantly altered patterns of precipitation,^35^ more lightning strikes,^36^ an expanding WUI^6^ and a history of fire suppression near communities^7^ fueled multiple fire seasons that were highly destructive and disruptive to human communities.^5^ Over the past two and a half decades, annual area burned has exceeded the historical frequency and extent for the region (Figure 1), which has correlated with an increase in unprecedented societal impacts. For example, the 2004 fire season in Alaska (USA), which was nearly seven-times larger by area than the previous 55-year average, was characterized by persistent, hazardous air quality.^37^^,^^38^ In Canada, the 2003 and 2011 fire seasons spurred costs well over 1 billion Canadian dollars and drove over 50,000 people to evacuate.^39^ The 2016 Fort McMurray Fire forced over 88,000 people to evacuate and had an estimated cost of 3.7 billion Canadian dollars, making it the most expensive natural disaster in Canadian history.^40^ The 2019 Swan Lake Fire was the costliest wildfire in the USA that year, and still ranks as Alaska’s most expensive.^41^ Canada’s 2023 fire season shattered records as 18.5 million ha burned; it doubled the previous record fire year in 1989^42^ and forced over 230,000 people to evacuate their communities.^43^ Smoke from those fires stretched across large portions of Canada and the contiguous USA, driving up the rate of asthma-related emergency department visits 17%.^44^ The human impacts from those fires emphasizes the need to reimagine our relationship with fire.

### The where, why, and how of fuel breaks

Land managers must make difficult decisions about where and how to install fuel breaks. In the USA, a committee of multiple federal land management agencies develops Communities at Risk lists for each state which rank municipalities based on perceived threat level and property value.^45^ Those assessments, along with how effective different municipalities are at advocating for fuels reduction projects in their community, determines where and how funding is allocated. In Canada, the resource allocation process for developing fuel breaks varies by province and territory. In Yukon, for example, resources are prioritized for communities that have completed a community wildfire protection plan. Local capacity, social license, public engagement, and the availability of funding from all levels of government also play a role in management decisions. Prioritization approaches have been widely adopted because of the high cost of installing and maintaining fuel breaks in the region, which is driven by equipment, personnel, and their transportation.^46^ Boreal regions of Alaska and Canada have a low population density and limited roadway development. Further, many communities are located off the road system and are accessible only by air or waterways in the summer. The combination of limited budgets, high costs, and logistical difficulties means that some rural and Indigenous communities, which are often at high risk from wildfires,^1^^,^^7^ may not receive adequate support or funding for fuel break projects.

**Figure 2:**
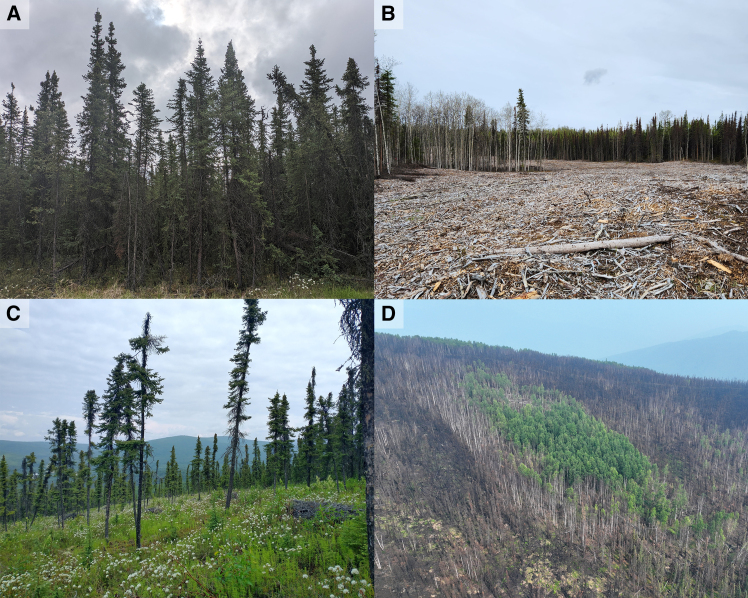
Multi-panel image showing fuel types, fuel breaks, and fire scars to add visual clarity to text in *The Where, Why, & How of Fuel Breaks* (A) *Picea mariana* stand, a typical fuel type of high management concern in the boreal forests of western North America due to the high degree of fuel connectivity. (B) Masticated fuel break near Sterling, Alaska, USA. (C) Thinned fuel break installed with chainsaws in Fairbanks, Alaska, USA. (D) Image from the 2021 Munson Creek Fire outside Fairbanks, Alaska, USA. Notably there is a live patch of *Betula neoalaskana* that survived the fire surrounded by dead *Picea mariana* that did not, demonstrating differences in plant flammability and vulnerability to fire. All photographs are courtesy of Nicholas Link and Michelle Mack.

When evaluating perceived wildfire threat levels for communities and other values, land managers are primarily concerned with reducing fuel loads in conifer forests composed of *Picea mariana* (black spruce), *Pinus contorta* (lodgepole pine), or *Pinus banksiana* (jack pine) that are prevalent in the region and support high rates of fire spread.^47^ Conifers contain highly flammable compounds in their needles and grow at high densities with a high degree of vertical and horizontal connectivity^48^^,^^49^ (Figure 2A). Fire spread from surface-to-crown is facilitated by ladder fuels and from crown-to-crown by high canopy foliar density. Fuel breaks are designed to disrupt the continuity of these conifer fuels and provide firefighters with access to support active management.^10^ To accomplish this, land managers can employ a variety of techniques, each of which has operational and ecological trade-offs. Mechanical treatments carried out with large equipment such as masticators, shear-bladers, or skid-steers can completely remove the canopy, the insulating soil organic layer, and the understory plant community (Figure 2B). While these treatments can greatly decrease fire intensity,^50^ they also have negative environmental impacts such as increased soil erosion or abrupt permafrost thaw and subsidence.^51^ Complete removal of the understory may also facilitate the establishment of flammable grasses on the newly exposed mineral soil.^50^ Installation of mechanical treatments relies on expensive equipment and qualified operators. In contrast, treatments where fuels are thinned with chainsaws disturb less of the canopy, soil organic layer, and understory plant community^15^^,^^52^^,^^53^ (Figure 2C). However, because there are remaining fuels, they require regular retreatment to remove regrowing conifers^17^ and some land managers do not find them to be operationally effective.^54^ Installation of hand-thinned treatments also has high per-unit-area labor costs^46^ and are thus not well suited for large projects. Evaluating ecological and socio-economic trade-offs in context is an important aspect of fuel break planning.^19^

The human and financial costs attributed to wildfires are expected to accelerate over the remainder of the century^3^ making it imperative that we adopt a battery of proactive measures including installing fuel breaks, building hardening, and changes to urban planning,^55^ to aid wildland firefighters in protecting communities. To that end, federal governments in Canada and USA have recently dedicated substantial financial support to wildfire protection efforts. Approximately 1.5 billion US dollars^56^ and over 269 million Canadian dollars^57^ have been allocated over the next five-year period to national fire protection efforts. It is crucial that those and other non-recurring financial resources are spent efficiently to help northern communities adapt to climate change. We propose that innovating on the design of fuel breaks so they provide co-benefits would limit trade-offs by adding ecological and socio-economic value, which would improve their capacity to reduce the long-term risks of wildfires to communities, enhance other climate adaptation efforts and increase social acceptance.

## Fuel breaks reimagined

Our effort to envision alternative designs for fuel breaks with co-benefits draws on the collaborative expertise of community members, ecologists; economists; social scientists; Indigenous Governments; and land managers from local, Territorial, and Federal agencies. We gathered public input on what co-benefits community members and land managers most desired through four listening and innovation sessions at regional meetings. Participants opted to attend our sessions that were offered at both regional environmental conferences and focused, local meetings on fuels related issues (supplemental information, Table S1). Fifty-seven attendees participated in our sessions out of their own interest and included local community members, as well as state, federal, Tribal, and non-profit agency employees. Attendees from 31 rural and urban communities in Alaska, Yukon, and the contiguous United States participated in a brainstorming exercise where they were given 15 min to sketch a map of their community, responding to the prompt (supplemental information, Figure S2):

“Draw a map of a future or current fuel break in your community with your ideas on how it could be used. Where should fuel breaks be placed and what should they look like? What plants and activities would you like to see in fuel breaks?”

Fifty-seven maps were returned with signed consent that they would be used to inform project development and future discussions of fuel break co-benefits. Forty-six percent of the participants were from rural communities, and 54% were from urban communities. The majority of participants were either employees of First Nations or Tribal Organizations (42.1%) or were university researchers or educators (26.3%). From the 57 maps, we identified 14 unique fuel break co-benefit themes, which we then ranked based on their frequency of occurrence.

**Figure 3:**
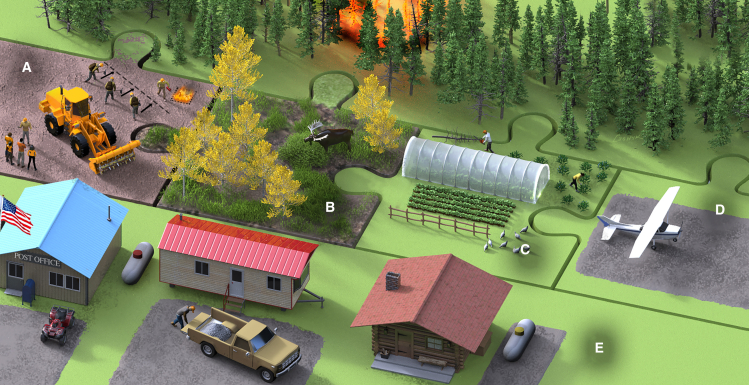
Conceptual visualization of each of the four broad fuel breaks scenarios in *Fuel Breaks Reimagined* Conceptual visualization of each of the four broad fuel breaks scenarios. Scenarios are shown as matching, interlocking pieces demonstrating that they are interchangeable and can be connected; communities can determine which scenario, or combination of scenarios, may work best for them depending upon their ecological and socio-economic context. (A) *Fire Focus*, showing firefighter training and fuel break research. (B) *Ecological Conversions*, showing a deciduous forest with native wildlife. (C) *Enhancing Food & Subsistence Resources* showing small scale vegetable production and forageable wild foods. (D) *Infrastructure & Recreation* showing a rural airstrip. (E) Community the fuel break is protecting. Original image by Victor Leshyk.

We evaluated and discussed these 14 themes as a group of interdisciplinary experts at the National Center for Ecological Analysis and Synthesis, generalizing them into four broad fuel break scenarios we deemed plausible within the ecological and socio-economic context of northern communities (Figure 3). Crucially, each of these scenarios maintains tactical use for wildfire operations as their primary purpose while supporting complementary activities that promote local co-benefits. For each scenario, we considered co-benefits as well as barriers and additional costs to implementation and their ecological and socio-economic trade-offs (Table 1). Further, we provide the rare few case studies of each scenario in practice to highlight innovative designs that have been undertaken across the region. The limited *in situ* examples demonstrate both their feasibility as well as their barriers and trade-offs. While not exhaustive of all potential options, these scenarios represent what we believe to be the most effective future directions for fuel breaks in the region.

**Table 1:** Summary of the main ecological and socio-economic co-benefits as well as barriers and trade-offs for the four fuel break scenarios at the end of *Fuel Breaks Reimagined*, following all scenarios

Scenario	Consideration	Co-benefits	Barriers and Trade-offs
Fire Focus	Ecological	Opportunities for ecological research	Large degree of intervention High level of disturbance
	Socio-economic	Potential for job opportunities Public education Training	Costly installation and maintenance Reduction in cultural services provided by ecosystem Long-term investment
Ecological Conversions	Ecological	Low degree of intervention Maintenance of natural ecosystem services	Minor level of disturbance
	Socio-economic	Low maintenance costs Maintenance of ecosystem services Integrated into the landscape	Cost of procuring seeds and seedlings Long-term investment May be ineffective at reducing fire behavior under extreme conditions
Enhancing Food & Subsistence Resources	Ecological	Maintenance of some ecosystem services	Large degree of intervention with some options Fertilizer and pesticide usage High level of disturbance with some options
	Socio-economic	Food security Potential for job opportunities Offsetting cost of installation and maintenance Public Education Passive maintenance	Costly installation and maintenance Expansion of the WUI Hesitancy around use as fuel break Potential for negative wildlife encounters
Infrastructure & Recreation	Ecological	Careful planning could reduce overall WUI footprint	Large degree of intervention High level of disturbance with some options
	Socio-economic	Potential for job opportunities Offsetting cost of installation and maintenance Improving quality of life Passive maintenance	Costly installation and maintenance Expansion of the WUI Hesitancy around use as fuel break Expectations that it would pay for itself

### Scenario 1: Fire focus

In this scenario, the main purpose of fuel breaks—wildfire risk reduction—is reemphasized and expanded to include fire management training, research, and education opportunities that increase self-efficacy for living with fire. Enthusiasm for fuel break installation is often greater than for fuel break maintenance. However, if a fuel break is not maintained (i.e., flammable vegetation is not regularly removed), its operational effectiveness is diminished. Fuel break maintenance is a key component of this scenario as they become permanent infrastructure that can support regular activities such as training new firefighters and practitioners, educating homeowners, demonstrating new techniques and equipment, burning for cultural or management reasons, and executing ecological or environmental research (Figure 3A). The community of Tok, AK has taken this approach by regularly re-treating the Red Fox fuel break and using that space as an education and demonstration site.^58^ Fuel breaks that are a dedicated component of a community’s wildfire protection plan could be designed for the aforementioned activities which foster fire adapted communities.

#### Co-benefits

Through ongoing engagement with fire management and research, fire protection is enhanced in this scenario. With regular maintenance, a community can ensure that access for equipment and personnel in a fuel break will always be possible when needed. Further, having a space for firefighter training and community education reinforces a culture of wildfire preparedness. Technical training and knowledge transfer are critical to sustain the human dimension of wildfire risk reduction. Fuel breaks designed with this intention could support large and small-scale experimentation to address operational issues (e.g., comparing the efficiency of equipment) or answer relevant ecological questions (e.g., the environmental impacts and burn rates in vary prescription types.^54^ Lastly, this fuel break scenario is the most effective way to get fire back on the landscape safely. In appropriate situations, training and research activities could support safe cultural and prescribed burns. Both the tribal non-profit organization Gwich’in Council International and the Arctic Council^59^ have advocated for reintroducing cultural burning as a crucial part of wildfire risk reduction, pest control, and wildlife management.

#### Barriers and trade-offs

Fuel breaks designed solely for fire risk mitigation likely have little to no ecological or economic co-benefits to the community. With a singular focus on wildfire management, there is little to temper the potentially large environmental footprint of management actions.^15^^,^^51^^,^^53^ In this scenario, fuel breaks are primarily a tool for the wildfire management community meaning they provide fewer co-benefits to the broader public. Given this, regular communication efforts with the public would be necessary to maintain engagement and create support for routine maintenance. Finally, there is an inherent fire risk associated with firefighter training and prescribed burning. Runaway burns severely harm public trust in the capacity of land management agencies to adequately reduce risk.^60^

### Scenario 2: Ecological conversions

Conifer fuels pose the greatest wildfire risk in the boreal forest. Of less concern are forests dominated by native deciduous broadleaf species, such as *Betula neoalaskana* (birch) and *Populus tremuloides* (aspen), which have functional traits that support lower flammability, slower fire spread, and low severity burning^2^^,^^47^^,^^61^^,^^62^^,^^63^^,^^64^ (Figure 2D). If fuel breaks were to reforest with native deciduous species, they could act as natural fire retardants (Figure 3B). Ecologists have promoted the idea of utilizing native deciduous stands as natural fuel breaks in the boreal forest for over a decade,^62^^,^^65^^,^^66^^,^^67^ and the Government of Yukon is testing this idea at scale in a fuel break southwest of Whitehorse, Yukon, Canada by planting 168,000 aspen over a two year period with another 265,000 planned for 2025 (*L. Bibeau, personal communication*). The ecological conversion concept can be extended to other native vegetation-types where appropriate, such as wetlands, grasslands, and shrublands as all vegetation-types have their own fuel characteristics that can be variably utilized by wildland firefighters.^68^

#### Co-benefits

Promoting native plant communities that have desirable fuel characteristics is a nature-based adaptation measure for the increasing threat of wildfires due to climate change.^69^^,^^70^ A “living” or “natural” fuel break made of native vegetation would not require much, if any, maintenance and its lifespan would be equal to the lifespan of the dominant species or seral stage. Further, if impacted by a wildfire, deciduous species such as aspen and birch have a high capacity to regain dominance after the disturbance.^71^ Natural environments provide ecosystem services (e.g., habitat for wildlife, climate regulation, carbon storage, water infiltration, cultural benefits, and so forth) without disrupting the viewshed. Fuel breaks designed in this way would seamlessly blend into adjacent unmanaged ecosystems, adding to landscape-scale heterogeneity. That aspect is important for Indigenous communities, such as Teslin, Yukon, Canada, where residents have expressed hesitancy to accept projects that are deemed harmful to the forests. In contrast, the people of Teslin are interested in promoting birch trees to create natural fuel breaks, as they have traditional uses for the wood, bark, and sap (*D. Cooley, personal communication*).

#### Barriers and trade-offs

A major hurdle with this scenario is that there is limited research on what controls revegetation post-treatment or how to best initiate ecological conversions. In mixed conifer and deciduous stands, promoting broadleaf trees may be as simple as selectively removing flammable conifers and allowing the broadleaf species to fill in the gaps. Conversely, in monospecific conifer stands, entire areas would likely need to be cleared and then replanted or reseeded with deciduous species. Another logistical hurdle is that there are few established markets to produce or sell local seeds or plants in the region. Those complications, in combination, make costs difficult to estimate. From a risk reduction perspective, all vegetation is flammable, especially in extreme conditions or during certain times of year.^72^ Changing disturbance regimes—including fire severity, drought related mortality, and insect outbreaks—can override species’ resistance to fire, weakening their capacity as a retardant. Additionally, natural vegetation may limit firefighter egress and thus be detrimental to operational efficiency. A final drawback is that reaching the desired endpoint of an ecological conversion would take decades. Therefore, commitment to the project by the community would need to be on a similarly long timescale.

### Scenario 3: Enhancing food and subsistence resources

Many communities in the boreal forest, especially those with Indigenous peoples, value hunting, trapping, gathering food and natural medicines, using wood for fuel and building, and other practices that use materials from the land. There is also an interest and need for agriculture.^73^^,^^74^ Fuel breaks can help to support these local food systems and other land-based practices by providing land for large and small-scale plant and animal agriculture. An example of small-scale agriculture is encouraging the re-growth or transplanting local vegetation, such as berries, into a thinned or cleared fuel break (Figure 3C). The Teslin Tlingit Council in Yukon has undertaken that approach by developing fuel breaks that provide fuel wood while encouraging revegetation by native plants that have traditional uses (*D. Cooley, personal communication*). Larger-scale agriculture development in Whitestone, AK, anchored fuel breaks to the community’s agricultural fields so that they could work in tandem for fire protection. That planning proved prescient as both the farm fields and the fuel breaks have been tactically important for fire crews twice in the last 25 years (*D. Rees, personal communication*).

#### Co-benefits

Supporting local food systems provides value for community health and well-being,^75^^,^^76^ cultural resurgence,^77^^,^^78^ and improved food security.^79^ Additionally, economic opportunities may develop from agricultural production, providing livelihoods to individuals and possibly offsetting the initial costs of the fuel break’s installation. This scenario, more so than the previous two, would give the public a sense of ownership of that space. Agricultural plots or foraging areas can be outdoor classrooms for sharing information on horticultural practices and Traditional Knowledge. Fuel breaks close to communities with traditional foods and medicines can also provide easier access to gathering areas for those with mobility issues or limited time to be on the land. As an operational advantage, using those spaces for production would also passively maintain their effectiveness as a fuel break. Human and livestock actions, including picking berries, tending garden plots, and grazing, would help retain low levels of flammable vegetation and high levels of firefighter egress.

#### Barriers and trade-offs

Any development within the fuel break is an expansion of the WUI. As fuel breaks are designed to be protective space, people may interpret them as a safe place to build. Once there are structures in a fuel break, such as hoop houses, chicken coops, or raised beds—firefighters may no longer perceive that area as a fuel break but as infrastructure that needs protection. A related issue is that if people have come to know a space for its food production, they may be resistant to its usage during wildfire operations. Regarding practical implementation, not every area that is best positioned for a fuel break would be possible to cultivate, given soil and landscape factors. Furthermore, if people want to grow local foods that they have traditionally foraged for, they may be challenged by procuring local seeds and seedlings. Considering the implications to the environment, this scenario could have a relatively low impact (e.g., promoting forageable, native plants) or a sizable environmental footprint (e.g., large-scale production) due to tillage and the application of fertilizers, pesticides, and herbicides.^80^^,^^81^ In the case of animal agriculture, there is a risk of promoting undesirable livestock-wildlife and human-wildlife encounters and the development of zoonotic diseases.^82^

### Scenario 4: Infrastructure and recreation

The final scenario focuses on embedding fire protection into community planning and development (Figure 3D). As communities in the boreal forest grow, they can develop with wildfire suppression in mind. Development in this context can either be for infrastructure (e.g., roads, airstrips, gravel pits, and so forth) or recreation (e.g., hiking/skiing trails, shooting ranges, sports fields, and so forth). Regardless of purpose, areas of development would be designed and situated for operational use by firefighters. For instance, developers could place a rural airstrip between the community and the natural area that poses the highest fire risk. Many communities already use their airstrips as firelines, whether they were intentionally placed for that purpose or not. Planning for recreation can similarly be tied into protection efforts. Mount Sima ski resort in Whitehorse, Yukon, Canada, is directly connected to the Copper Haul Road fuel break, allowing fire crews to use it in emergency situations. As communities plan development, it would be valuable to explore how growth can reduce their wildfire risk.

#### Co-benefits

The greatest advantage of this scenario is that fire protection can be synergistically incorporated into project planning, with co-benefits that improve cost efficiency, social acceptance, and project longevity. Development that is designed to improve the overall quality of life through infrastructure or recreational opportunities is likely to have more community engagement and greater support for initial investment and cost of maintenance. Consistent usage would ensure that these areas are well maintained as a fuel break. Moreover, some projects would remove all vegetation, which is of notable value for fire operations. Another added advantage is that many pieces of infrastructure have tactical utility (e.g., Mount Sima’s snow making equipment can quickly transport water). This scenario has the greatest potential to be financially self-supported. If an area is designed for recreation, it could generate revenues that support jobs and help subsidize installation costs. Infrastructure projects can be public-private partnerships, as opposed to being completely tax-payer funded. Leveraging private sector capital may be an effective way to support the costly development of fuel breaks.

#### Barriers and trade-offs

The drawbacks of this scenario are parallel and amplified versions of many of the drawbacks in the *Enhancing Food & Subsistence Resources* scenario. Any development is an expansion of the WUI, so firefighters would unquestionably need to protect permanent infrastructure. All development has significant environmental impacts (e.g., paved roadways have low infiltration rates and are inhospitable for most plants). Not all places that are ideal for building are well positioned for fire protection (e.g., the placement of airstrips needs to prioritize safety for takeoffs and landings above all else). There is also the potential issue of community resistance to its usage as a fireline during emergency situations. Navigating bureaucracy, high costs, and conflicts between competing stakeholder groups are additional challenges. Development can be controversial, and public-private partnerships can lead to costly and time-intensive disputes.^83^

## Implementing sustainable solutions

**Figure 4:**
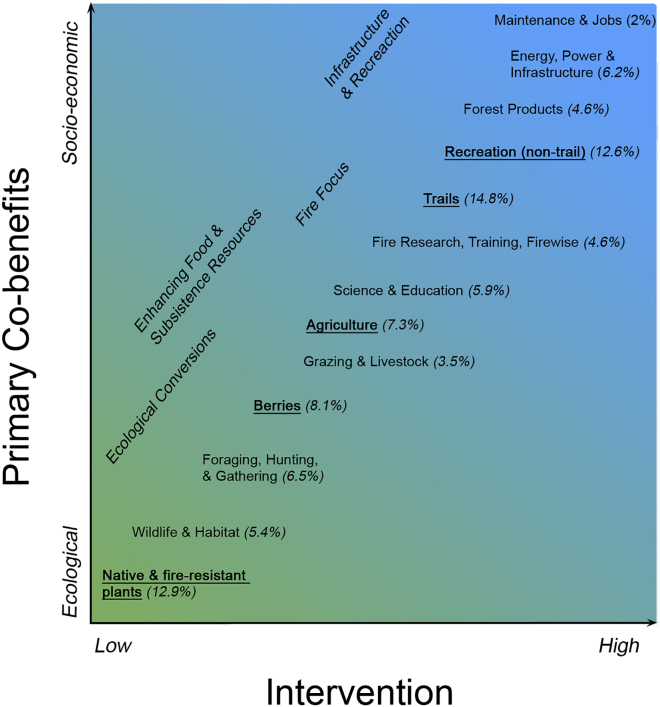
Fuel break endpoints plotted on two axes (degree of human intervention and primary co-benefits) in *Implement Sustainable Solutions* Broad themes from each fuel break scenario placed along axes of human intervention (i.e., how far they diverge from natural conditions) and a spectrum of co-benefits, from primarily ecological to primarily socio-economic. Themes came from our interactive listening sessions with land managers and community members. Themes that are bolded and underlined were the top five most often listed during those sessions. How often each theme was listed (as a percentage) in parentheses next to the theme name. Only endpoints that were listed more than 2% of the time are shown.

No one fuel break design will fit all communities. Ecological and socio-economic context can provide a framework for determining what is possible and appropriate. The scenarios we have proposed exist along a spectrum that ranges from ecological to socio-economic co-benefits. This spectrum is positively related to the degree of human intervention required (i.e., the degree to which they alter natural ecosystem processes and structure) (Figure 4). Placing co-benefits along these axes can enable communities to identify which scenario best aligns with their values, and available time and resources. For example, a community with socio-economic priorities may choose co-benefits that fall under *Infrastructure and Recreation* but will have to acknowledge that this requires a greater degree of intervention to build and maintain. We note that the top five most frequently identified themes in the fuel break maps from the listening session spanned from low intervention with ecological co-benefits to high intervention with socio-economic co-benefits. We also note that each scenario can also be placed along these axes and can be implemented in multiple ways (e.g., the *Enhancing Food & Subsistence Resources* scenario encompasses both *Foraging, Hunting, & Gathering* as well as *Grazing & Livestock*) (Figure 4). Communities will have to prioritize strategies based upon needs, values, and resources.

**Figure 5:**
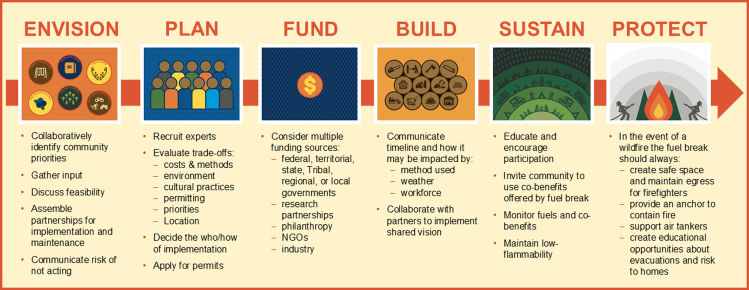
Conceptual roadmap in *Implement Sustainable Solutions* Conceptual roadmap for community integration of co-benefits into boreal forest fuel breaks. Graphics courtesy of Jen Gunderson.

Real barriers exist to applications of each scenario. Addressing challenges and improving our understanding of context will require a collaborative and community-engaged approach. To help in that effort, we have developed a roadmap that leads community groups through envisioning, planning, funding, building, and sustaining a fuel break designed to protect and provide for their community (Figure 5). To enrich the roadmap, we created a workshop facilitation plan that includes real life examples, planning cards that detail co-benefits available in each scenario, and a community-based fuel break mapping exercise (supplemental information). We developed these tools so that communities can easily discuss including co-benefits in the community wildfire protection plan process, which can be important to access some streams of funding.^84^

## Next steps for implementation and research

A key concern for many communities in the region is preserving the natural structure and function of the local environment. In situations where environmental concerns are paramount, land management and policy efforts should rely on nature-based solutions, including directed ecological conversions toward *Native & Fire-Resistant Plants* or the promotion of native *Berries* in fuel breaks. To enact the nature-based scenarios we have outlined, communities will need adequate access to native plant materials that are locally sourced, as well as ecological research on the best ways to establish native plants. A current impediment is that there are not yet established large-scale forestry or horticultural industries in the region with this knowledge, and ecological research on native plant propagation is limited. To develop local solutions, existing community organizations, land management agencies, local stewards, and academic researchers will need to collaborate on those efforts. Those collaborations may be most effective if they draw upon Indigenous and local ways of knowing along with western-style scientific research in a Two-eyed Seeing approach.^1^^,^^85^^,^^86^^,^^87^

Funding is a persistent issue and has long been the primary limiting factor for land management agencies. Many of the economic challenges posed, however, can be reframed as opportunities.^88^ Each scenario could create jobs (e.g., tree planters and ski instructors) or spur economic development (e.g., local tree and horticultural nurseries, tourism). Future research will be needed to determine the best ways to maximize those opportunities as well as identifying situations where public-private partnerships or development could offset the costs of fuel breaks. In situations where all costs cannot be offset, cost-benefit analyses and studies on willingness-to-pay can inform the viability of fuel break projects. Economic research can support land managers to combat the effect of “crowding out,” where public investment in fuel breaks can decrease the level of private risk mitigation.^89^ Outcomes from those investigations are certain to highlight differences between communities, as priorities vary across the region.

Social and cultural implications of fuel breaks are crucial to consider and are best incorporated by collaboratively developing co-benefits with local communities and other relevant parties. We echo the call from Christianson et al. (2022) that Indigenous Peoples should be able to make decisions about their own territories and be included in region-wide planning, as they see fit. Social science research can support collaborative planning dialogues as well as the resulting on-the-ground actions.^73^^,^^90^ As interest in fuel break co-benefits grows, there are opportunities for research that supports knowledge sharing among northern communities to improve the success of all treatments. This can also extend to global knowledge sharing and solidarity building with communities around the world that take similar approaches to climate change and wildfire adaptation.^91^

## Conclusions

Wildfires in the boreal forests of western North America are a growing threat to people as communities expand and the climate changes. We need to adopt a cohesive suite of preventative measures to mitigate risk, and fuel breaks are a critical component of that multi-pronged approach. Given the relatively short history of fuel breaks in the region, there is room for innovation in their design so that they offer a more complete complement of ecological and socio-economic co-benefits. Planning of fuel breaks is central to efficient and effective use of this tool, and we stress the need for future research to enhance that process. Those interdisciplinary investigations can help tailor fuel breaks to the values and resources of the communities they serve. Continued collaboration between land managers, scientists, Indigenous representatives, economists, government agencies, and the public is necessary to ensure that fuel breaks are developed and installed to best meet the diverse needs of communities. Active incorporation of co-benefits into fuel break design can help ensure that these spaces remain effective and maximally useful to communities. As recent fire seasons in the region have demonstrated, adaptation to changing fire risk needs to be on every local agenda.

## Acknowledgments

This work resulted from the Sustainable Fuel Breaks Working Group, part of the Morpho Initiative led by JFJ and MCM and conducted at the 10.13039/100014585National Center for Ecological Analysis and Synthesis at the 10.13039/100007183University of California, Santa Barbara, with support from the 10.13039/100000936Gordon and Betty Moore Foundation as part of their Wildfire Resilience Initiative. Community listening sessions were supported through a fire planning grant from 10.13039/100000001NSF (OPP-2332346) to JFJ, MCM, and KVS. Additional support was provided by the Bonanza Creek Long-Term Ecological Research (BNZ-LTER) program, with funding from 10.13039/100000001NSF (DEB-2224776 and DEB-1636476) and the 10.13039/100006959USDA Forest Service, 10.13039/100013393Pacific Northwest Research Station to MCM. NTL, MCM, and XJW were supported by an 10.13039/100000001NSF Navigating the New Arctic grant (RISE-2127284) to MCM and XJ.

## Author contributions

M.C.M. and J.F.J. conceived the project and organized and led the working group. All authors contributed at the working group sessions to develop the fuel break scenarios. J.F.J, K.V.S., and E.E.S. ran the community listening sessions. N.T.L. wrote the article under the supervision of M.C.M. and J.F.J. All authors edited and reviewed the article.

## Declaration of interests

The authors declare no competing interests.
