# Supplementary material for: Mitigating increasing wildfire risk through fuel break innovations
Source: iScience. 2025 Dec 10;29(1):114391. doi: 10.1016/j.isci.2025.114391 (PMC12803937; doi:10.1016/j.isci.2025.114391)
Supplement: Document S1. Figures S1 and S2 and Table S1 [file mmc1.pdf]

iScience, Volume *29*

Supplemental information

Mitigating increasing wildfire risk

through fuel break innovations

Nicholas T. Link, Jill F. Johnstone, Xanthe J. Walker, Felecia Amundsen, Hazel K. Berrios, Luc Bibeau, Dorothy Cooley, Ann C. Erickson, Carla Johnston, Joseph M. Little, Nathan Lojewski, Alison D. Perrin, Carly A. Phillips, Stefano Potter, Daniel C. Rees, Lisa B. Saperstein, Jennifer I. Schmidt, Emily E. Sousa, Katie V. Spellman, Andrew Spring, and Michelle C. Mack

Supplement 1

Public Listening Sessions

Participants were drawn from attendees at larger conferences and meetings who selected our session out of their own interest (Table S1). The participants included local, state, federal, Tribal and non-profit agency employees interested in wildfire management and planning. Fifty-seven participants in our workshop were from 31 different communities, primarily from Yukon Territory and Alaska, but five were from communities in the contiguous US states. We did not collect demographic data beyond the community that the participant was from and their organization or affiliation. Nearly half of the participants (46%) were from rural communities, and 54% were from urban communities. A plurality of participants were employees of First Nations or Tribal Organizations (42.1%) and over a quarter were university researchers or educators (26.3%) (Fig. S1). The remainder of participants worked for State, Territorial, or Federal agencies, local or regional non-profits, K-12 schools, their own businesses, or had no employment listed (Fig. S1).

Table S1: Name, date and location information for the four conferences where we recruited attendees for our listening sessions

Conference	Location	Dates
Whitehorse Fuels Breaks Workshop	Whitehorse, Yukon Territory, CA	February 22, 2024
Alaska Forum on the Environment	Anchorage, Alaska, USA	February 6, 2024
Alaska Wildland Fire Coordinating Group Fuels Meeting	Fairbanks, Alaska, USA	February 27, 2024
Alaska Tribal Conference on Environmental Management	Anchorage, Alaska, USA	March 26, 2024

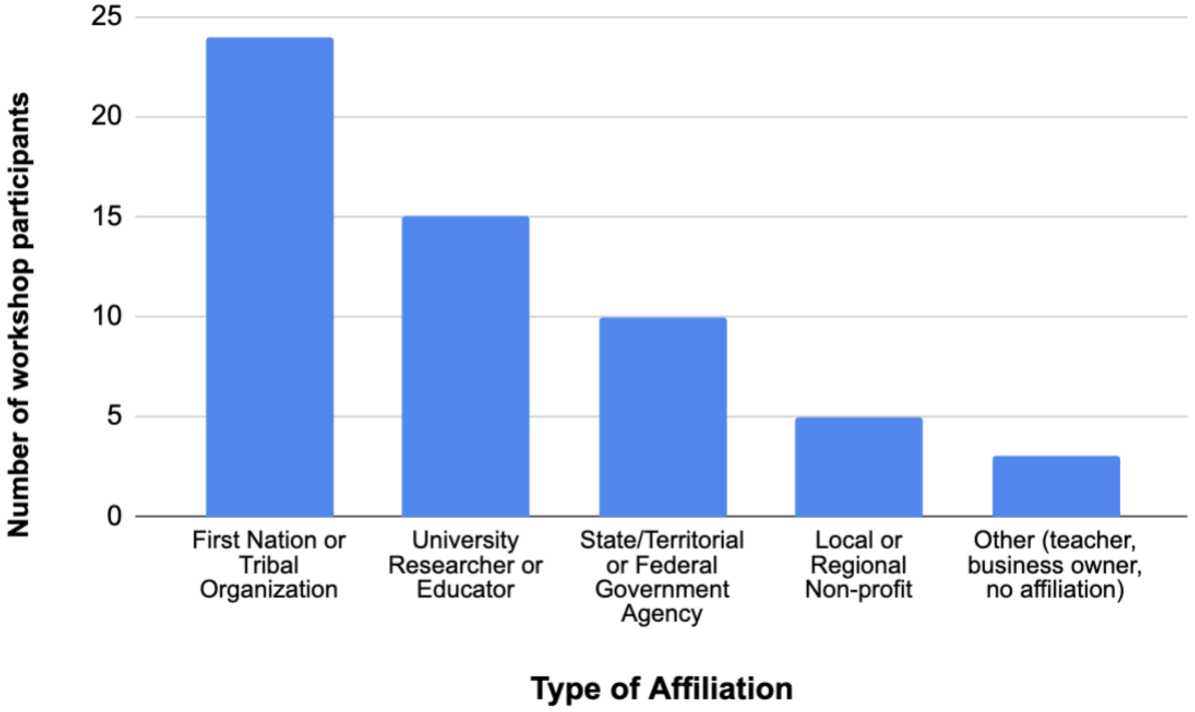

Figure S1: Workshop participant affiliation information.

Each session was 70-90 minutes in length. The sessions began with a brief introduction to the project and the concept of designing fuel breaks with co-benefits for communities. Participants were then given 15 minutes to draw a map of their community and sketch the benefits that they would desire in a fuel break they already have, or that they are planning. The data collection tool (Fig. S2) was a sheet of paper with the title of the workshop session and an informed consent checkbox and signature line. The prompt for the drawings—given orally, projected in the room, and stated—was as follows:

> *"Draw a map of a future or current fuel break in your community with your ideas on how it could be used. Where should fuel breaks be placed and what should they look like? What plants and activities would you like to see in fuel breaks?"*

Fifty-seven maps were returned by participants across the four workshop sessions. All participants who wished to share their drawing provided signed consent and were informed in writing that the drawing may be used in reports. The participants then shared their maps in small groups and self-identified common themes across the maps that had been drawn of fuel breaks and the benefits for their community that were desired. These themes were shared back to the larger group to close out the session.

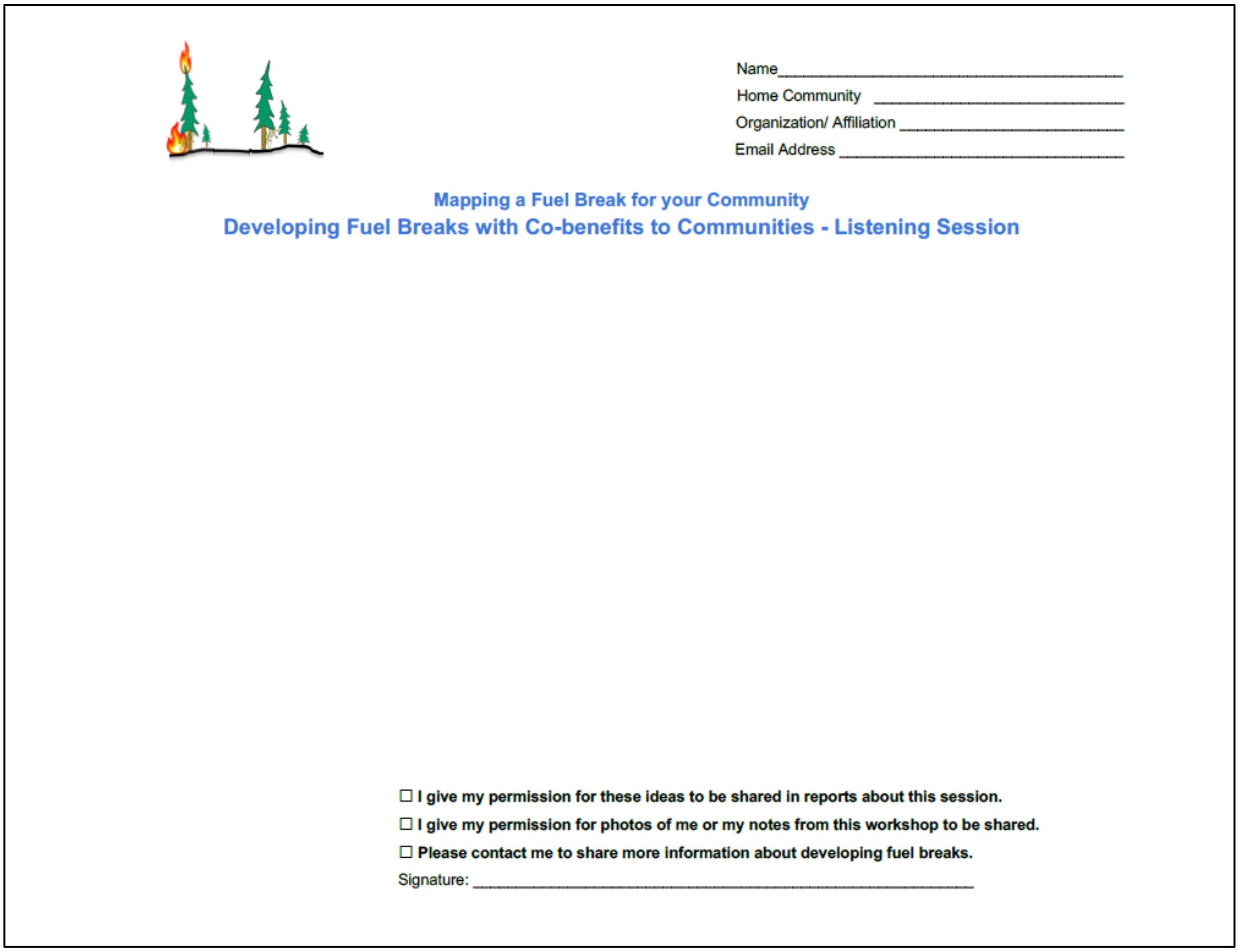

Name____________________________________
Home Community ___________________________
Organization/ Affiliation __________________________
Email Address ______________________________

Mapping a Fuel Break for your Community

Developing Fuel Breaks with Co-benefits to Communities - Listening Session

☐ **I give my permission for these ideas to be shared in reports about this session.**
☐ **I give my permission for photos of me or my notes from this workshop to be shared.**
☐ **Please contact me to share more information about developing fuel breaks.**
Signature: ____

Figure S2: Data collection tool used in the four public listening sessions.

Supplement 2

Table of Contents

Workshop Facilitation Guide……………………………………………………………………….1

Community Benefit Tokens for Mapping Fuel Break Activity……………………………………….5

Fuel Breaks with Benefits Scenario Planning Cards………………………………………………….6

Fuel Break Use Case Handouts……………………………………………………………………….11

Fuel Break Planning Roadmap Booklet……………………………………………………………….17

WORKSHOP FACILITATION GUIDE

Envisioning Fuel Breaks with Benefits

WORKSHOP GOALS

- Provide opportunity for discussion about fuel breaks and how they can offer **additional, desirable benefits** that promote community well-being.
- **Map out a preliminary vision** for an existing or future fuel break with additional benefits built into them.
- Introduce **next steps** for planning a fuel break with additional community benefits.

SUPPLIES NEEDED

- Large chart paper and markers
- *Community Benefit Tokens* (pre-cut)
- *Fuel Breaks with Benefits Scenario Planning Cards*
- *Fuel Break Use Case* handouts
- *Fuel Break Planning Roadmap* booklets
- Sticky notes

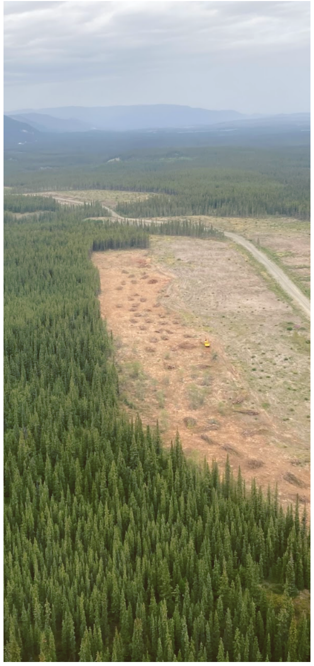

Wildfire fuel break in Whitehorse, Yukon. Photo: L. Bibeau

INTRODUCTION

Across the boreal North, communities are facing increasing wildfire risk due to drier fuels, longer fire seasons, and more extreme fire weather. Many communities in Alaska and Northern Canada lie within the wildland-urban interface, making them particularly vulnerable to wildfire. Fuel breaks that remove flammable vegetation can help reduce risk of wildfire and provide access for firefighters to help slow fire spread. Fuel breaks come in many forms, from shaded fuel breaks with some trees remaining to full removal of vegetation. The new spaces created, if intentionally designed, can offer additional benefits to communities.

Imagine ecological benefits such as revegetation with less flammable, native plant species; cultural and subsistence uses like berry patches or enhanced moose habitat; or economic and social opportunities through wood harvesting, local food production, or recreation spaces. Integrating community-defined values into the design can transform fuel breaks into broader community assets.

How do you get started creating a vision for your fuel break with benefits? The following workshop structure is designed for a **90-minute session** with flexibility to adjust to local contexts. It draws on facilitation methods successfully piloted in Alaska and Yukon listening sessions.

Big Ideas

What are wildfire fuel breaks? Strategically thinned or cleared areas designed to slow or stop the spread of fire have proven to be an effective wildfire risk reduction tool. They provide anchor points for firefighting, improve suppression access, and reduce the likelihood of catastrophic loss.

What do we mean by additional benefits? Additional benefits are positive secondary effects that result from a solution that primarily addresses a different problem. Often found in climate change and sustainability efforts, they are "win-win" situations where a single policy or project can achieve multiple simultaneous goals.

WORKSHOP PLAN

1. Welcome and Introductions (10 min)

- Introduce facilitators and the goals of the session. Have all participants introduce themselves.
  Tip: Keep introductions short and warm. Encourage participants to say their name, community, and why they care about fire and land stewardship.
- Emphasize the purpose of fuel breaks, which is wildfire protection, but that additional benefits can also be planned to enhance the value to the community.
- Provide a few visual examples to spark imagination (e.g., shaded fuel breaks, community gardens, berry patches, recreation spaces).

2. Introduction to Fuel Breaks (10 min)

- Explain what a fuel break is and how it works in fire management.
- Show slides or photos of fuel breaks in action to stop wildland fires and protect communities.
- Hand out examples of additional *benefit use cases* for participants to read more (e.g., Fairbanks fuel break used for berry picking, Anchorage shaded fuel break used for recreation and education, Teslin fuel break used for wood harvesting and traditional foods and medicines).
- Discuss how standard fuel break designs can be expanded to include additional benefits such as recreation, cultural use, food systems, or ecological conversions to less flammable forest.

Key message: *Fuel breaks can be more than just an empty space to reduce wildfire risk; they can be living parts of community landscapes that promote other aspects of community well-being.*

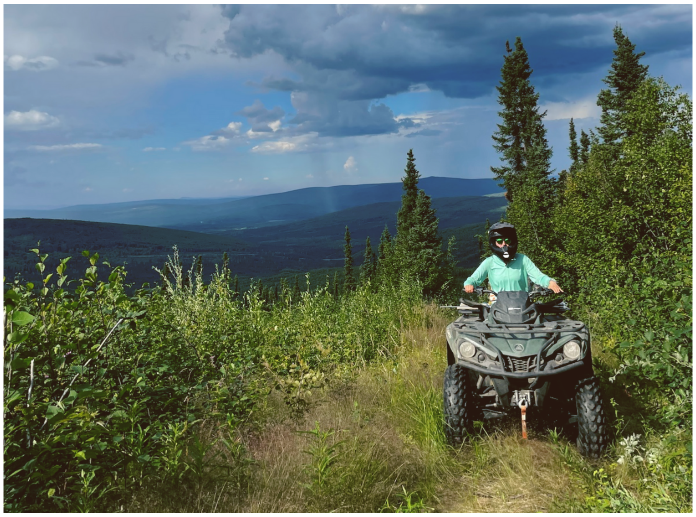

A fuel break on Murphy Dome in Fairbanks, Alaska has been used for ATV trails. It is also a popular berry picking area. *Photo: F. Amundsen*

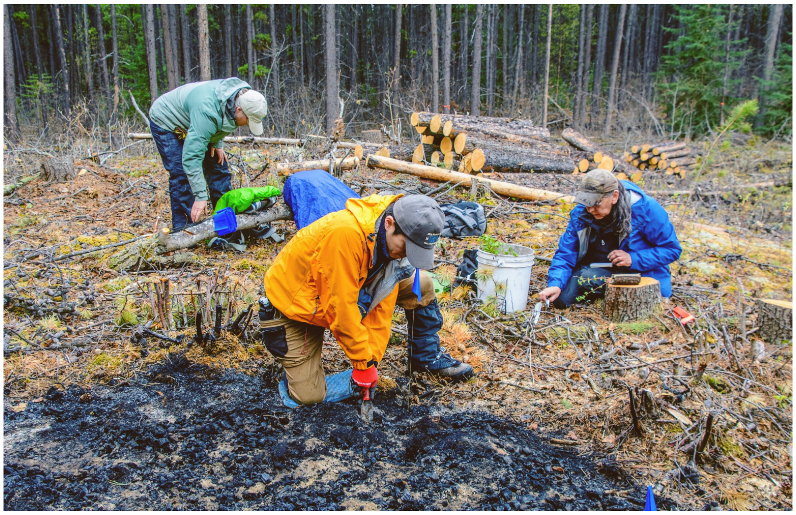

In Teslin, Yukon, community members have planned the wood harvesting area to also be a fuel break and are planting berries in the area. *Photo: J. Johnstone*

3. Fuel Break Mapping Exercise (30 min)

- Divide the group into smaller **groups of 3-5 people**. If you are providing this workshop with people from several communities, divide the participants by community.
- Distribute large chart paper, markers, *Fuel Break Benefit Scenario Planning Cards*, and *Community Benefit Tokens* among the groups.
- Ask participants to **draw a map** of their community on the large chart paper, then have the groups imagine a current or future fuel break in or near their community. Provide the following guiding prompts:

  - Where should the fuel break be placed on your map?
  - What should it look like? What plants or activities would you like to see there?
  - How could it serve both fire protection and other community needs?
  - Think big! Don't be limited by budget or bureaucracy.

- **Draw the fuel break on the map,** and use the Fuel Breaks with Benefits Scenario Planning Cards to discuss which benefits people want and where they could be in the fuel break. Which areas would be the most suitable for the different activities or benefits? Which are accessible to youth, elders or other community groups?
- Use the Community Benefit Tokens to physically **place different benefits or ecosystem services onto the map**. Glue or tape them in place on the map.

Tip: Consider where the most effective location would be for a fuel break, including wind direction and the location of the most flammable vegetation. The Alaska Wildfire Exposure tool might be useful to look at during this phase, and can be found at https://alaskanrm.com/web-apps/. Wind direction information for in Alaska can be found at https://snap.uaf.edu/tools/airport-winds.

4. Report Out and Gallery Walk (30 min)

- To facilitate each group sharing the ideas they came up with, have the whole group walk from table to table in a gallery walk.
- Each group presents the map and additional benefits they envisioned from their discussions.
- Encourage participants to use sticky notes to add affirmations, questions, or new ideas to each group's work.

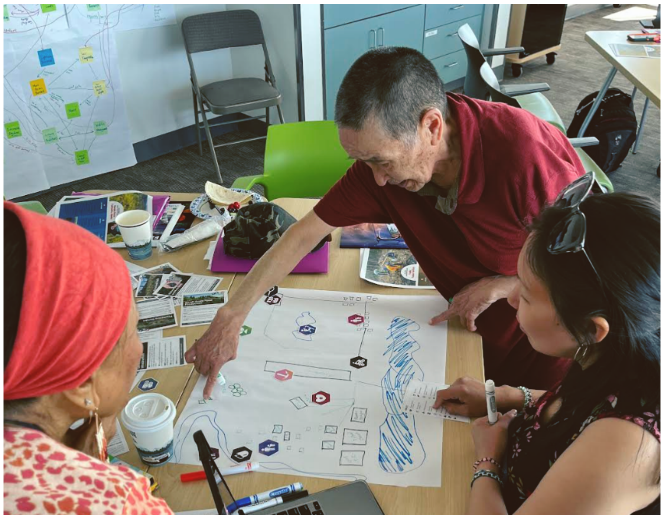

Mapping a fuel break with benefits during a workshop. *Photo: K. Spellman*

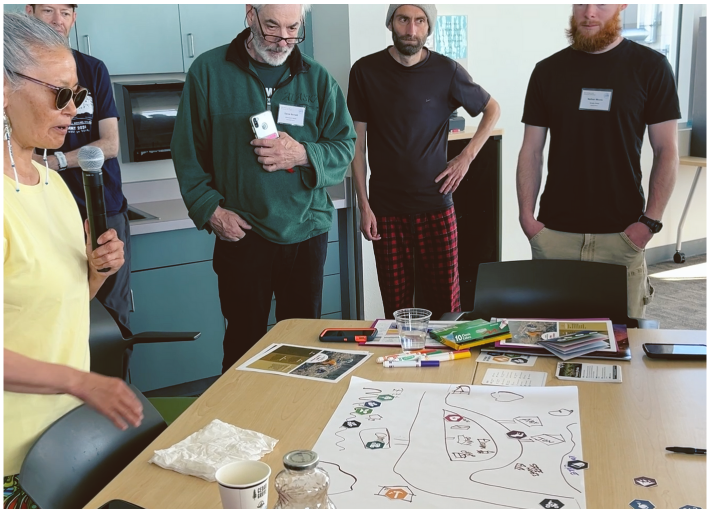

Sharing mapped fuel breaks with benefits to the larger group during a gallery walk. *Photo: K. Spellman*

5. Next Steps and Closing (10 min)

Envisioning a fuel break is just the beginning. To move from workshop ideas to setting groups in motion for implementation, discuss the implementation roadmap:

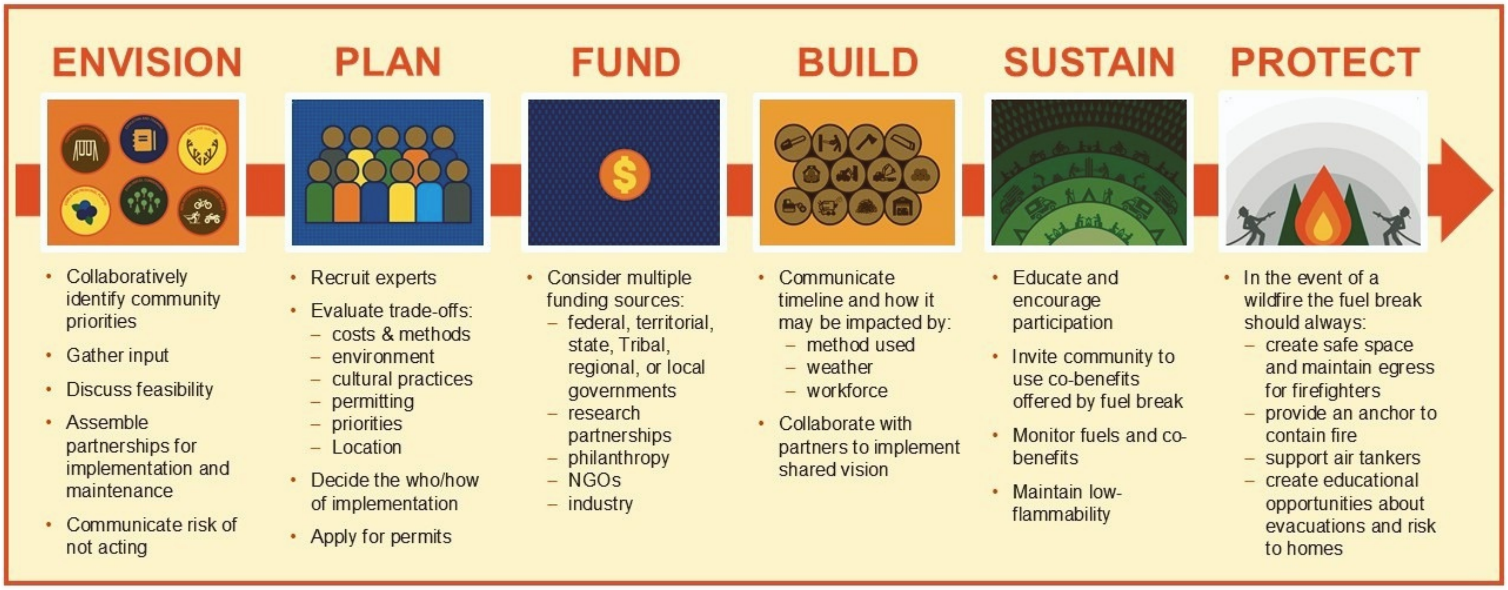

Along with this process consider:

- **How will you document and share** outcomes of the visioning process with your community and relevant agencies?
- **Who will be the champions** — local organizations, tribal governments, land managers — who can carry the vision forward?
- **How can this vision align** with wildfire risk reduction goals, Community Wildfire Protection Plan, cultural values, and funding opportunities?
- **Which of the desired fuel break benefit options is the best match** for the soils, vegetation, and site conditions, while maintaining the needed access for firefighters?
- **What other partnerships are needed** across jurisdictions, since many fuel breaks cross multiple land ownership boundaries?
- **What are funding options** (e.g., fire management, agriculture, habitat restoration) to increase feasibility of carrying out the vision?
- **How can you maintain community engagement** so fuel breaks remain valued, cared-for, and functional over time while maintaining low flammability?

The process is more fully outlined in the *Fuel Break Planning Roadmap* booklets, which should be distributed to all the workshop participants. By combining fire risk reduction with ecological, cultural, and economic benefits, communities can transform fuel breaks from just emergency measures into broader, long-term assets that strengthen resilience and well-being.

This workshop plan was developed by Katie Spellman, Emily Sousa, Jill Johnstone, Michelle Mack, and Nick Link. Graphics in the roadmap were developed by Jen Gunderson. This project is funded through support from the National Center for Ecological Analysis and Synthesis Morpho Initiative (PIs Jill Johnstone and Michelle Mack) and by the National Science Foundation FIRE-PLAN project "Social-Ecological Engineering and Design of Sustainable (SEEDS) Boreal Forest Fuel Breaks" (PI Jill Johnstone, Co-PIs Katie Spellman and Michelle Mack, Award # OPP 2332346). Additional support is provided through the Bonanza Creek Long Term Ecological Research Program (NSF DEB-2224776 and DEB-1636476) and by the USDA Forest Service, Pacific Northwest Research Station (RJVA-PNW-20-JV-11261932-018). The University of Alaska Fairbanks is accredited by the Northwest Commission on Colleges and Universities. UAF is an affirmative action/equal opportunity employer, educational institution and provider and prohibits illegal discrimination against any individual. Learn more about UA's notice of nondiscrimination.

Community Benefit Tokens

for Mapping Fuel Breaks Activity

Instructions:
Print and cut out each hexagon, which represents a benefit or ecosystem service that could be a part of your fuel break design. Place the tokens on the map of your community and fuel break. Move them around on the map as you consider the site conditions and the accessibility.

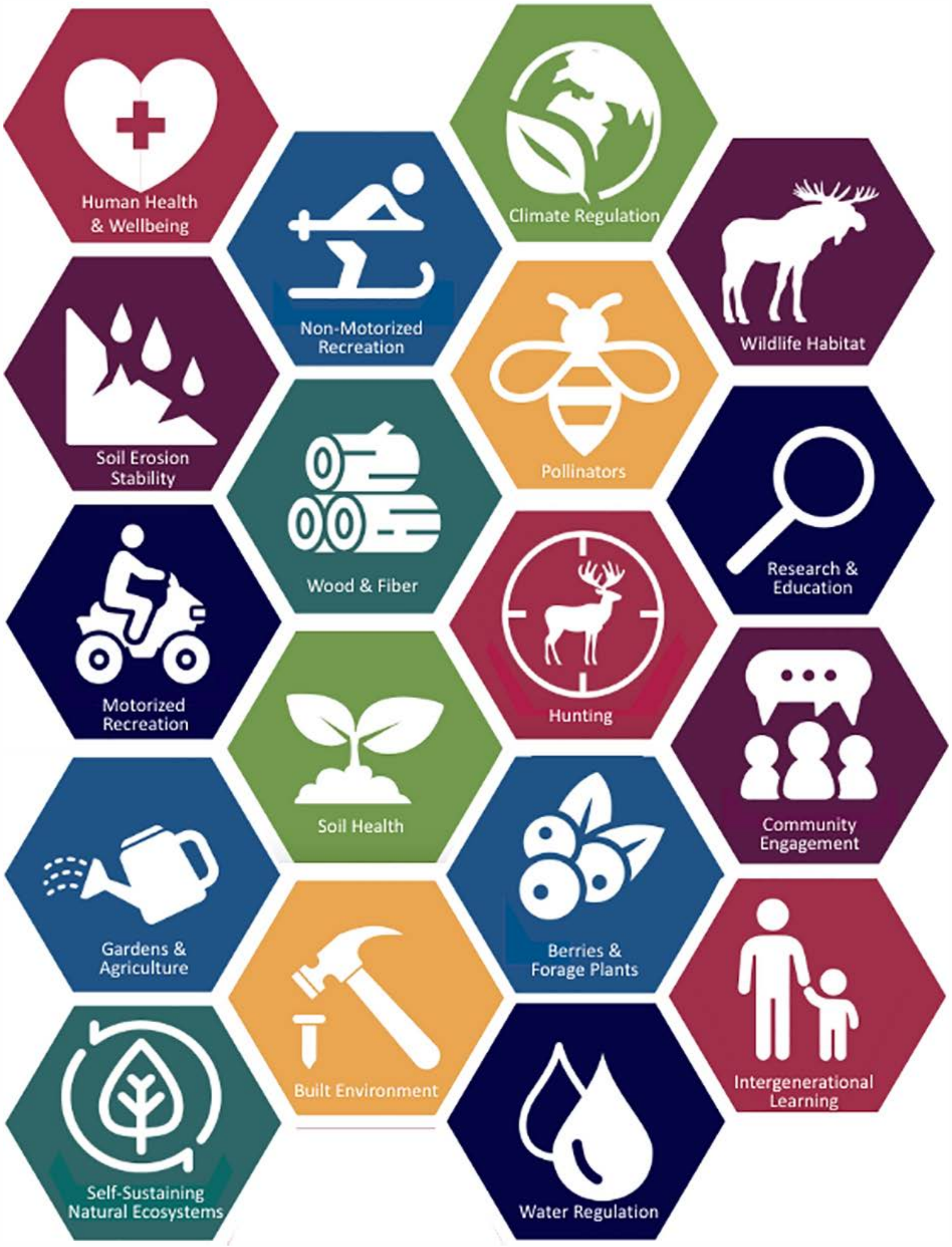

FUEL BREAKS WITH BENEFITS

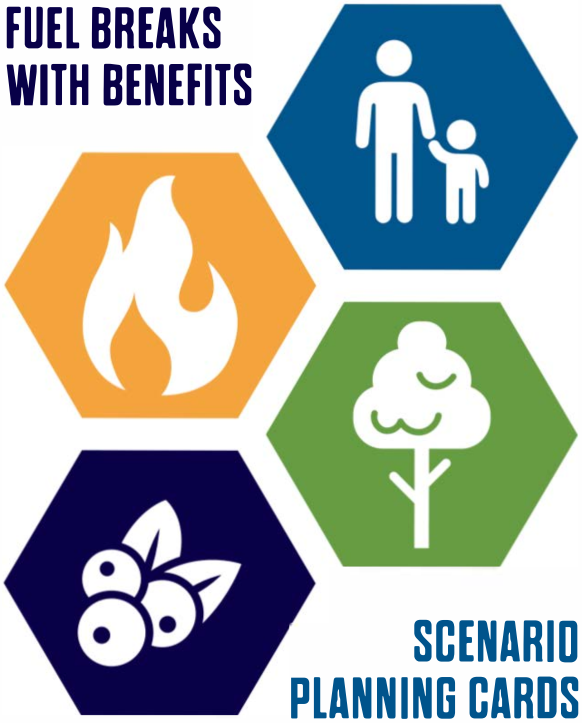

SCENARIO PLANNING CARDS

The "Fuel Breaks with Benefits" project acknowledges support from the National Center for Ecological Analysis and Synthesis (Morpho Initiative), US National Science Foundation (OPP 2332346), Bonanza Creek Long-Term Ecological Research Program, and University of Alaska Fairbanks.

GRAPHIC ATTRIBUTION:

Non-motorized recreation icon by Ayub Irawan

Motorized recreation icon by Andy Horvath

Hunting icon by Pekosman

Wildlife habitat icon from Freepik.com

Self-sustaining natural systems icon from Freepik.com

Built environment icon by Vectorslab

Intergenerational learning icon by Gukhyun Cho

Human Health & Wellbeing, Berries & Forage Plants, Research & Education, Soil Erosion Stability, Pollinators, and Community Engagement icons by Molly Putman, Geophysical Institute Design Services.

UAF is an affirmative action/equal opportunity employer, educational institution and provider and prohibits illegal discrimination against any individual: www.alaska.edu/nondiscrimination.

ICON KEY:

- Human health & wellbeing
- Non-motorized recreation
- Motorized recreation
- Community engagement
- Hunting
- Research & education
- Wood & fibre
- Climate regulation
- Soil erosion stability
- Soil health
- Wildlife habitat
- Self-sustaining natural systems
- Built environment
- Gardens & agriculture
- Intergenerational learning
- Berries & forage plants
- Pollinators
- Water regulation

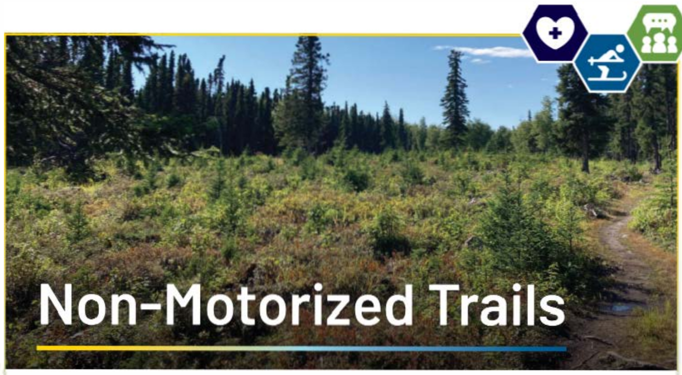

Photo credit: Nathan Lojewski.

Non-Motorized Trails

WHAT Provide non-motorized recreation opportunities.

WHY Reduce wildfire risk AND provide trails for skiing, biking, hiking, and other non-motorized recreation for exercise and transportation that will not create noise pollution.

WHERE Areas where noise reduction is a priority (near residential area) and soils are firm/dry.

HOW Build trail through thinned or cleared fuel breaks.

EXAMPLE Campbell Tract, Anchorage, Alaska.

HUMAN HEALTH & WELLBEING • NON-MOTORIZED RECREATION
COMMUNITY ENGAGEMENT

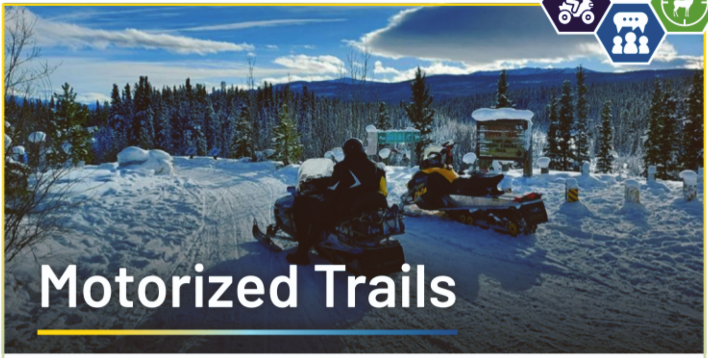

Motorized Trails

Photo credit: Klondike Snowmobile Association

WHAT Provide motorized recreation opportunities.

WHY Reduce wildfire risk AND provide trails for snowmobile/ATV, access for hunting, and other motorized recreation and transportation.

WHERE High fire risk areas where noise and conflict between user groups are not a concern. Areas where permafrost, erosion, and wildlife disturbance are not a concern.

HOW Connect cleared fuel breaks with access.

EXAMPLE Copper Haul Road, Whitehorse, Yukon.

MOTORIZED RECREATION • COMMUNITY ENGAGEMENT • HUNTING

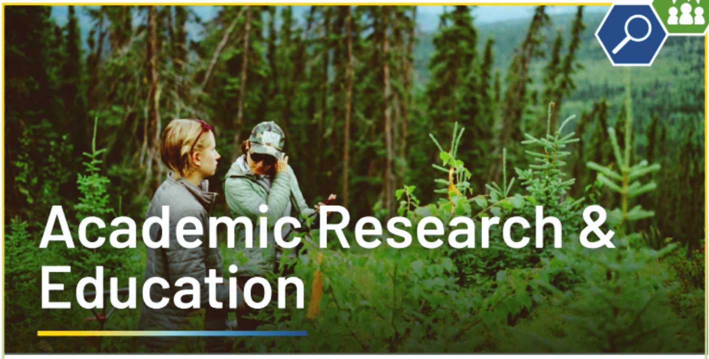

Academic Research & Education

Photo credit: Wesley Smith

WHAT Plant ecology research and education.

WHY Reduce wildfire risk AND provide opportunities for plant ecology research, testing plants responses to fuel treatment below ground and above ground changes, and expanding knowledge.

WHERE Areas that address knowledge gaps.

HOW Collaborate with universities or other partners.

EXAMPLE Fairbanks, Alaska.

RESEARCH & EDUCATION • COMMUNITY ENGAGEMENT

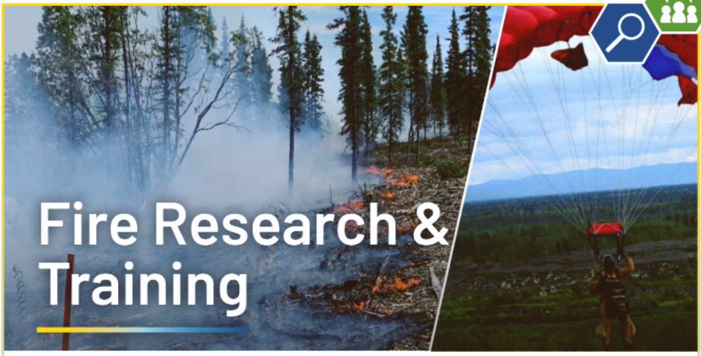

Fire Research & Training

Photo credit: Luc Bibeau, Maureen Clark

WHAT Fire science research, training, and education.

WHY Reduce wildfire risk AND provide opportunities for fire science research, testing fuel treatment efficacy, and expanding training and education.

WHERE Areas suitable for activity.

HOW Collaborate with universities or other partners. Collaborate with residents, management agencies, and research entities for risk reduction.

EXAMPLE Multiple locations.

RESEARCH & EDUCATION • COMMUNITY ENGAGEMENT

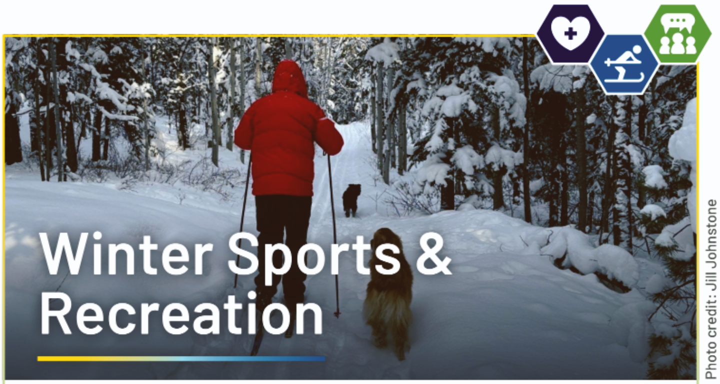

Winter Sports & Recreation

Photo credit: Jill Johnstone

WHAT Provide areas for winter sports, e.g. cross-country and downhill skiing, luge track.

WHY Reduce wildfire risk AND provide new opportunities for sports and recreation, potential training for professional sports.

WHERE Cleared or thinned fuel breaks near communities. Locations suitable for activity.

HOW Clear or thin fuel break. Collaborate with local recreation groups and/or schools.

EXAMPLE Whitehorse, Yukon; Anchorage, Alaska.

HUMAN HEALTH & WELLBEING • NON-MOTORIZED RECREATION
COMMUNITY ENGAGEMENT

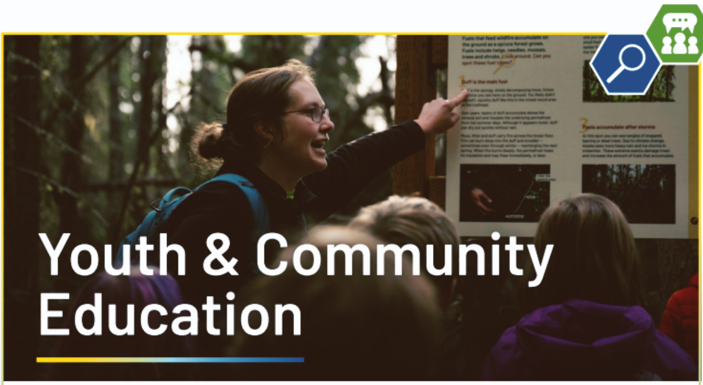

Youth & Community Education

Photo credit: Chynna Lockett

WHAT Ecological and wildfire education for the public.

WHY Reduce wildfire risk AND provide opportunities for youth and community members to learn more about their local ecosystem and the role of fire.

WHERE Areas suitable to the activity.

HOW Collaborate with schools, education or environmental non-profits, universities or other partners.

EXAMPLE Fairbanks, Alaska.

RESEARCH & EDUCATION • COMMUNITY ENGAGEMENT

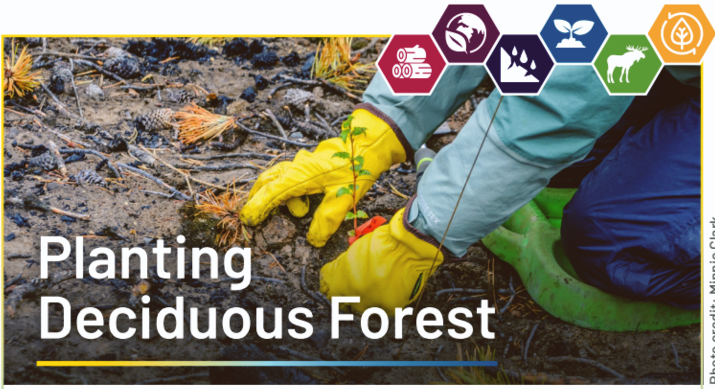

Planting Deciduous Forest

Photo credit: Minnie Clark

WHAT Deciduous tree planting to replace conifers.

WHY Reduce wildfire risk AND support wildlife habitat, support local food system, prevent erosion, stabilize and build soil, decrease flooding, store carbon.

WHERE Thinned or cleared areas with limited or no deciduous component in the forest stand.

HOW Conifer removal followed by tree planting.

EXAMPLE Whitehorse, Yukon and Teslin, Yukon.

WOOD & FIBRE • CLIMATE REGULATION • SOIL EROSION STABILITY • SOIL HEALTH
WILDLIFE HABITAT • SELF-SUSTAINING NATURAL SYSTEMS

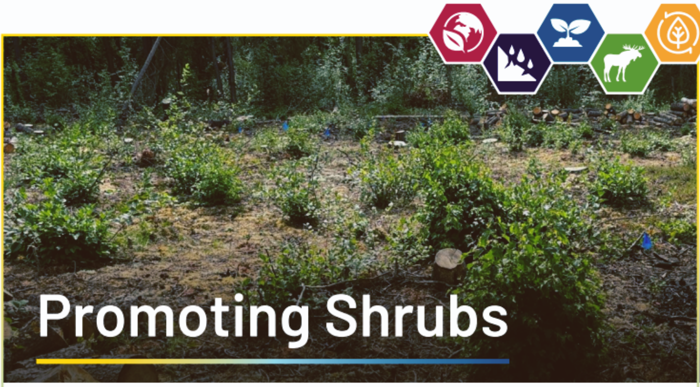

Promoting Shrubs

Photo credit: Jill Johnstone

WHAT Conversion to shrubland.

WHY Reduce wildfire risk AND support wildlife habitat, provide food and fuel, prevent erosion, stabilize and build soil, decrease flooding, store carbon, deter trespassing.

WHERE Moderate to dry soils, flat ground and hill slopes.

HOW Conifer removal with soil scarification followed by natural seeding, or retain existing shrubs.

EXAMPLE Teslin, Yukon.

CLIMATE REGULATION • SOIL EROSION STABILITY • SOIL HEALTH WILDLIFE HABITAT
SELF-SUSTAINING NATURAL SYSTEMS

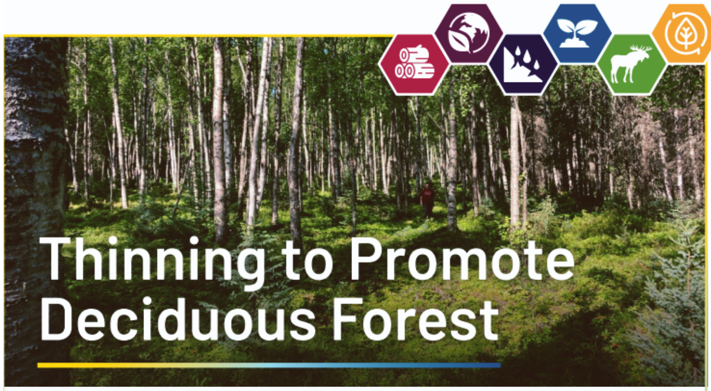

Thinning to Promote Deciduous Forest

Photo credit: Nathan Lojewski

WHAT Thin to promote deciduous forest.

WHY Reduce wildfire risk AND support wildlife habitat, provide food and fuel, prevent erosion, stabilize and build soil, decrease flooding, store carbon.

WHERE Forests with a mix of conifer and deciduous trees.

HOW Conifer removal with deciduous retention.

EXAMPLE Campbell Tract, Anchorage, Alaska.

WOOD & FIBRE • CLIMATE REGULATION • SOIL EROSION STABILITY • SOIL HEALTH
WILDLIFE HABITAT • SELF-SUSTAINING NATURAL SYSTEMS

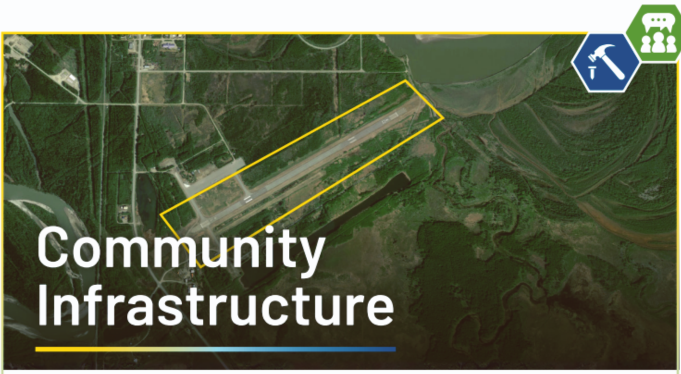

Community Infrastructure

Photo credit: Google Earth

WHAT Strategically place infrastructure that requires clearing, e.g. roads, airstrips, sports fields, playgrounds, grey water drainage, septic lagoon.

WHY Reduce wildfire risk AND enhance community well-being and/or services.

WHERE Close to communities, area suitable for infrastructure

HOW Clear fuel break, collaborate with residents and management agencies.

EXAMPLE Nenana, Alaska.

BUILT ENVIRONMENT • COMMUNITY ENGAGEMENT

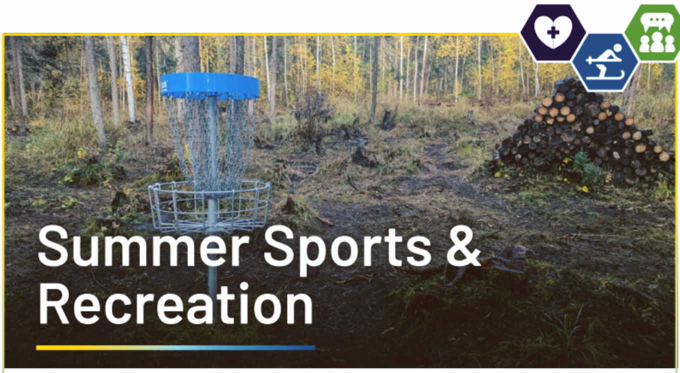

Summer Sports & Recreation

Photo credit: UDisk, Crocus Bluff Disc Golf Course

WHAT Provide areas for summer sports, e.g. disc golf fairways, golf course, driving ranges, sports fields, gun range.

WHY Reduce wildfire risk AND provide new opportunities for sports.

WHERE Cleared or thinned fuel breaks near communities.

HOW Clear or thin fuel break. Collaborate with local recreation groups and/or schools.

EXAMPLE Crocus Bluff, Dawson City, Yukon.

HUMAN HEALTH & WELLBEING • NON-MOTORIZED RECREATION
COMMUNITY ENGAGEMENT

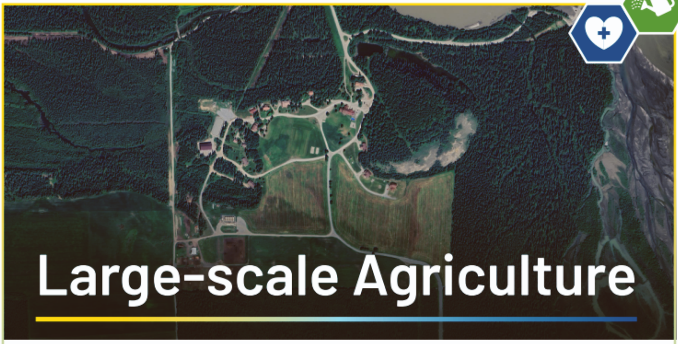

Large-scale Agriculture

Photo credit: Google Earth

WHAT Large-scale agriculture and/or livestock grazing.

WHY Reduce flammability AND support local and market food systems, potential economic opportunities, forage for livestock.

WHERE Locations with good soils, close to communities.

HOW Clear forest, prepare soil for agricultural planting, collaborate with food producers.

EXAMPLE Whitestone, Alaska.

HUMAN HEALTH & WELLBEING • GARDENS & AGRICULTURE

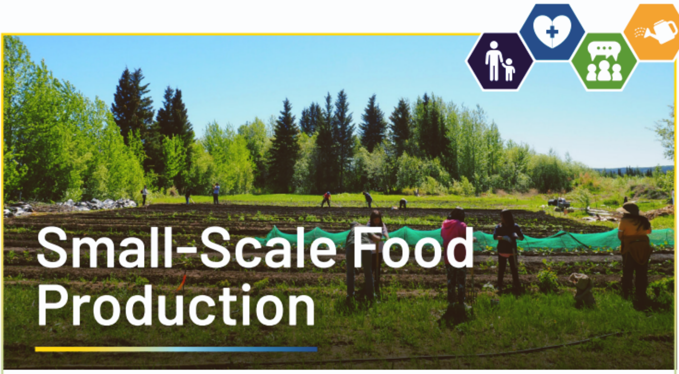

Small-Scale Food Production

Photo credit: Carla Johnston

WHAT Small-scale agro-ecological growing and/or appropriate livestock.

WHY Reduce flammability AND support local food system and livelihoods, prevent erosion, stabilize and build soil.

WHERE Locations with good soils, close to communities.

HOW Clear or thin forest, prepare soil for planting, use local compost and other inputs if possible, collaborate with local food producers.

EXAMPLE Sambaa K'e, Northwest Territory.

INTERGENERATIONAL LEARNING • HUMAN HEALTH & WELLBEING
COMMUNITY ENGAGEMENT • GARDENS & AGRICULTURE

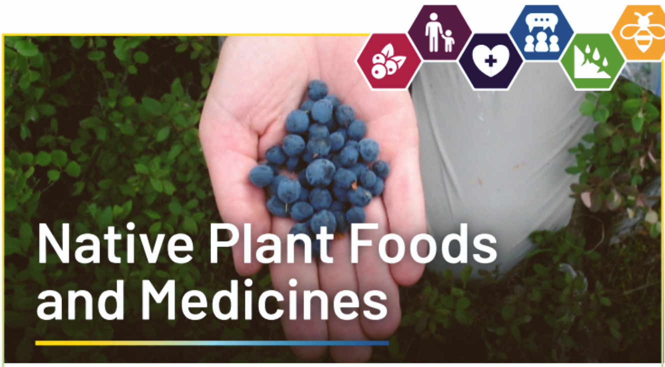

Native Plant Foods and Medicines

Photo credit: Katie Spellman

WHAT Manage native vegetation for food, medicine, fuels and other uses.

WHY Reduce flammability AND support local food system and livelihoods, accessible gathering locations, opportunities for intergenerational knowledge transfer, support wildlife habitat, prevent erosion, stabilize and build soil.

WHERE Easily accessible to community, soils suitable for desired vegetation.

HOW Clear or thin forest, provide conditions to promote existing desired species and/or to transplant from another part of the bush.

EXAMPLE Kakisa, Northwest Territories; Teslin, Yukon.

BERRIES & FORAGE PLANTS • INTERGENERATIONAL LEARNING • HUMAN HEALTH & WELLBEING • COMMUNITY ENGAGEMENT • SOIL EROSION STABILITY • POLLINATORS

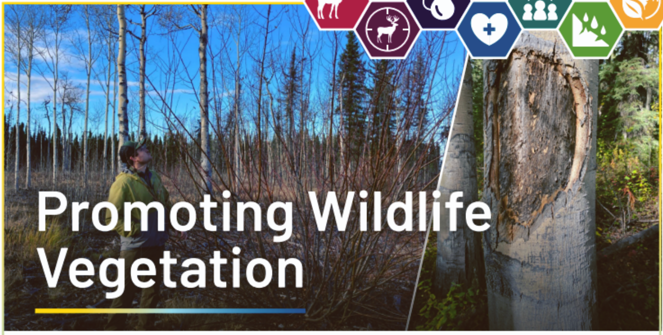

Promoting Wildlife Vegetation

Photo credit: Nathan Lojewski, Dorothy Cooley

WHAT Regenerate vegetation for wildlife food species, e.g. aspen for moose browse.

WHY Reduce flammability AND support wildlife habitat, support local food system, prevent erosion, stabilize and build soil, decrease flooding, store carbon.

WHERE Soils that support desired vegetation.

HOW Methods suitable for desired vegetation (e.g. masticate or roller chop to promote hardwood regeneration), collaborate with local hunters and trappers.

EXAMPLE Sterling and Tok, Alaska.

WILDLIFE HABITAT • HUNTING • WATER REGULATION • HUMAN HEALTH & WELLBEING • COMMUNITY ENGAGEMENT • SOIL EROSION STABILITY • CLIMATE REGULATION

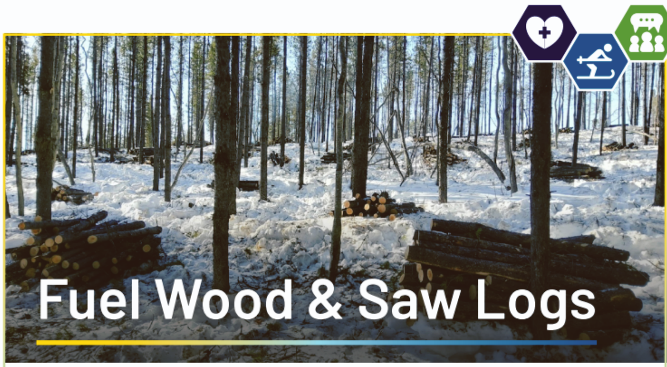

Fuel Wood & Saw Logs

Photo credit: Luc Bibeau

WHAT Thin fuels and use cut wood for firewood, biomass burners, or saw logs.

WHY Reduce flammability AND support local wood needs and livelihoods while providing accessible harvesting locations.

WHERE Areas easily accessible to the community, soils suitable for harvesting and gathering wood

HOW Clear or thin forest, stockpiling wood for local use.

EXAMPLE Yukon FireSmart locations.

HUMAN HEALTH & WELLBEING • COMMUNITY ENGAGEMENT
WOOD & FIBRE

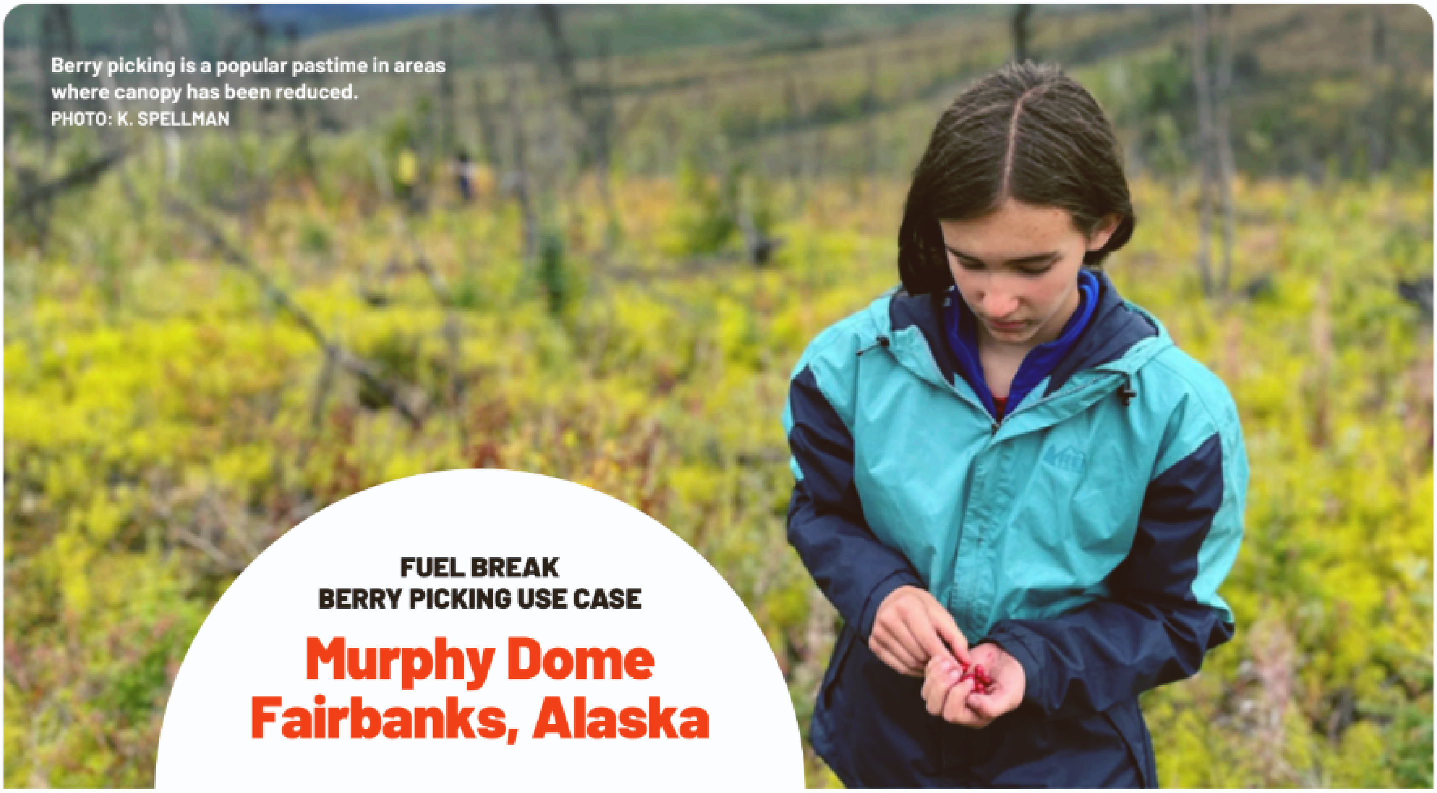

Berry picking is a popular pastime in areas where canopy has been reduced.
PHOTO: K. SPELLMAN

FUEL BREAK
BERRY PICKING USE CASE

Murphy Dome Fairbanks, Alaska

WHAT WAS DONE

After back-to-back record breaking fire seasons in 2003-2005 that caused several neighborhoods to be evacuated in the outlying areas of Fairbanks, the State of Alaska Department of Forestry, Fairbanks Office partnered with the Fairbanks North Star Borough to establish a Community Wildfire Protection Plan. The construction of the Old Murphy Dome Road and Murphy Dome and Spinach Creek Roads Fuel Break was completed in 2009. The fuel break was designed to protect the northern outlying community of Fairbanks, AK. It is 600+ acres of a linear shear bladed fuel treatment about 100 m wide.

HIGHLIGHTS

- The Murphy Dome fuel break protected homes during the 2019 Shovel Creek Fire. An estimated $51 million of homes and buildings were within 5 miles of the fire perimeter.
- The area has been an attractive berry picking, hunting and recreation area for decades, and the fuel break has provided improved berry habitat and recreational access.

CO-BENEFITS

- Berry picking area the is extended beyond the alpine habitat of the top of Murphy dome.
- Increased cross-country skiing and winter trail access, increased visibility for summer ATV trail.
- Summer goat and livestock grazing for neighborhood residents.

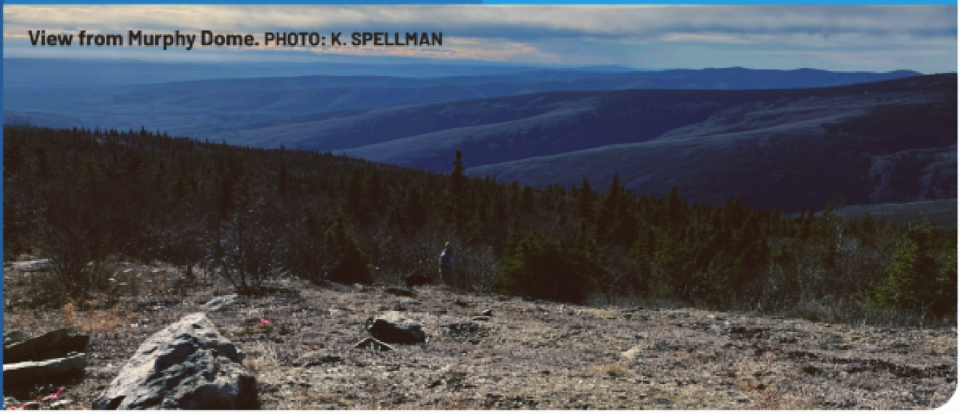

View from Murphy Dome. PHOTO: K. SPELLMAN

WHO STARTED IT?

- 2003-2005 fires forced the preparation of the 2006 Fairbanks North Star Borough Community Wildfire Protection Plan (CWPP).
- 2003 Healthy Forest Restoration Act (HFRA) directed communities to complete risk and mitigation plans for wildfire.

WHAT WAS THE INTENTION?

- Protect outlying areas of Fairbanks from wildfire.
- Murphy Dome has been used as a popular recreation area for hunting, berry picking and northern lights viewing, since the road was established in 1950s for military operations (DEW line).

Below: The Murphy dome fuel break protected homes during the Shovel Creek Fire in 2019.
PHOTO: ALASKA DEPARTMENT OF FORESTRY

HOW DID THEY DO IT?

The habitat was primarily black spruce forest. They created a 600 acre shear bladed fuel break with complete stand removal.

WHO WAS INVOLVED?

Planning: The Fairbanks office of the State of Alaska Division of Forestry led the project.

Implementation: Contractors were hired to clear the land.

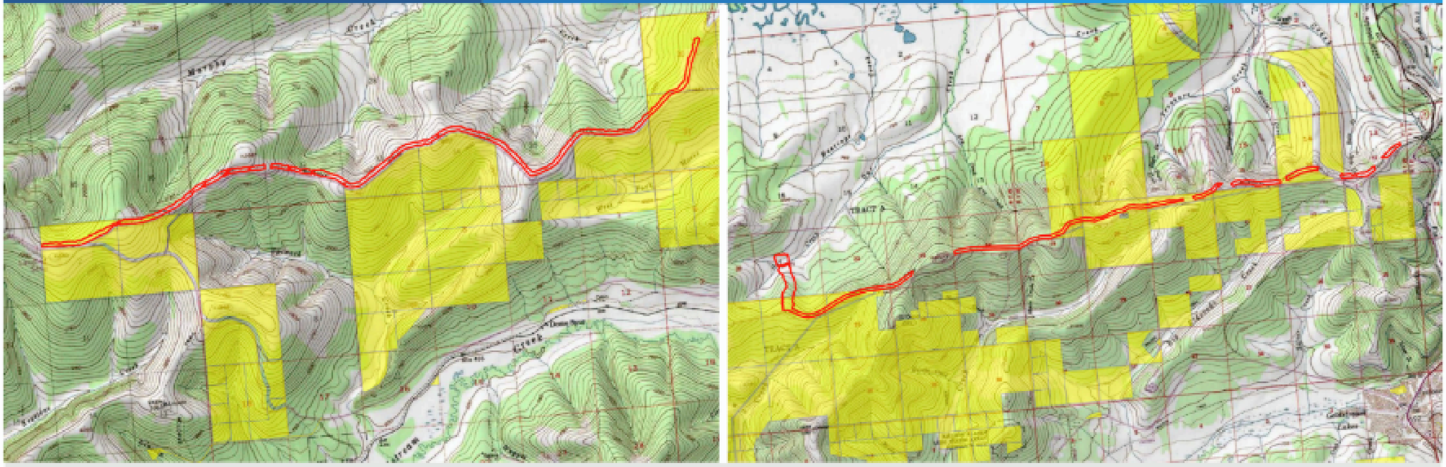

Murphy Dome Fuel Treatment Increases Berry Production in *Vaccinium vitis-idaea*

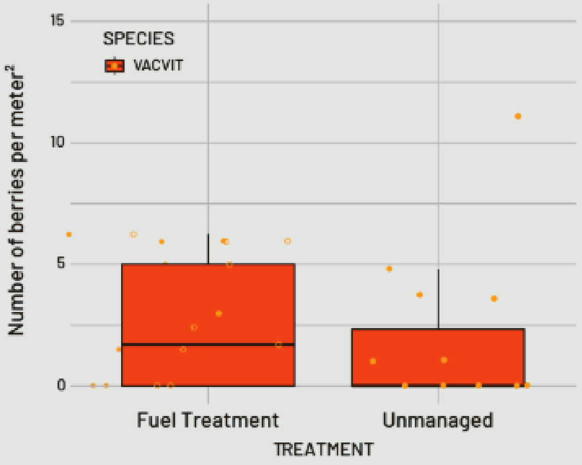

Murphy Dome Fuel Treatment Increases Berry Production in *Vaccinium uliginosum*

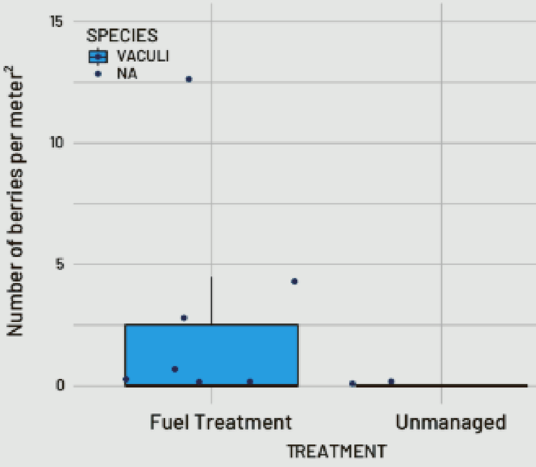

Research in the Murphy Dome Fuel Treatment shows that there is a higher density of blueberries and cranberries in the fuel break than in the adjacent forest.
GRAPHS: F. ADMUNDSEN, BONANZA CREEK LTER

The "Fuel Breaks with Benefits" project acknowledges support from the National Center for Ecological Analysis and Synthesis (Morpho Initiative), US National Science Foundation (OPP 2332346), Bonanza Creek Long-Term Ecological Research Program, and University of Alaska Fairbanks.

UAF is an affirmative action/equal opportunity employer, educational institution and provider and prohibits illegal discrimination against any individual: www.alaska.edu/nondiscrimination.

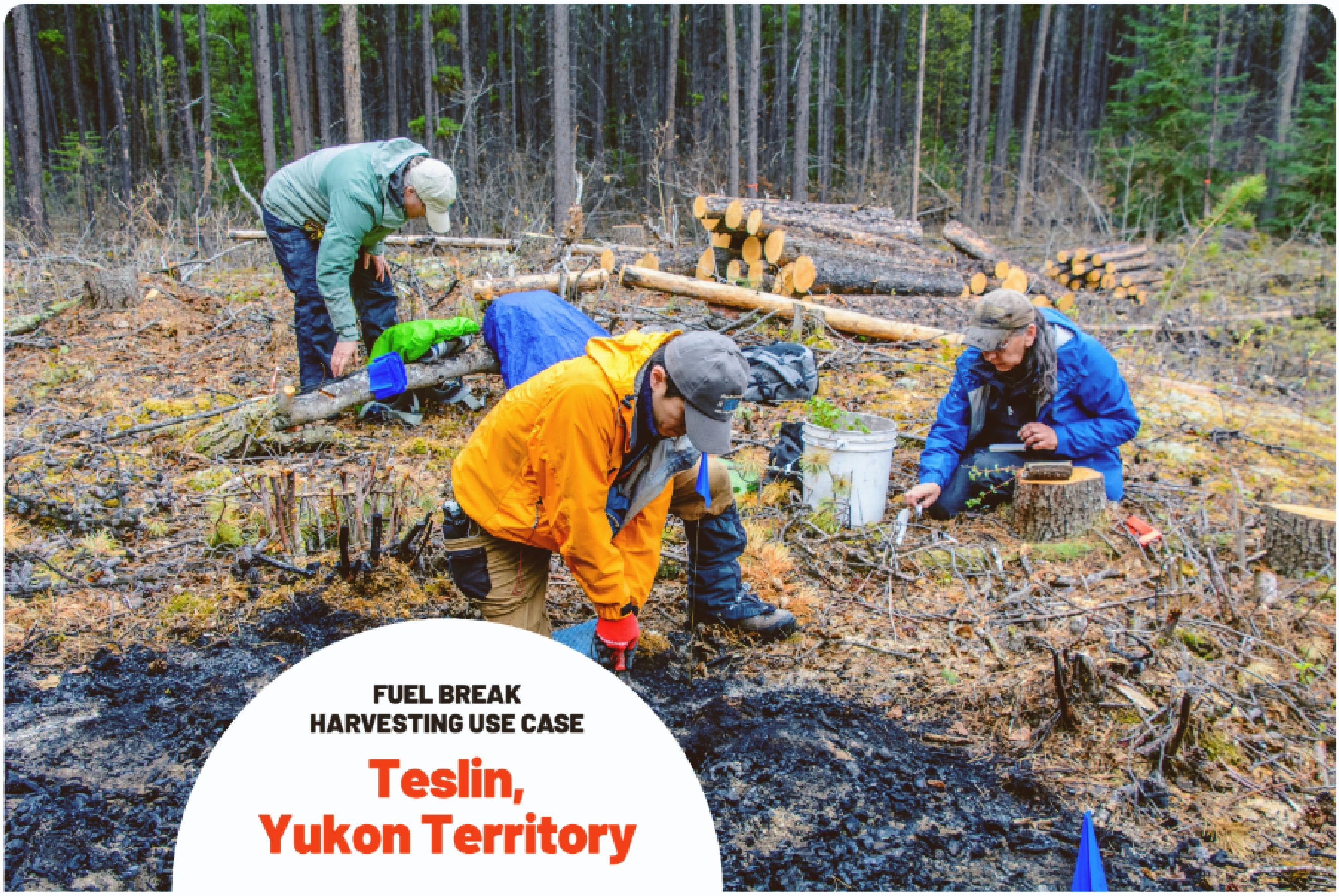

FUEL BREAK HARVESTING USE CASE

Teslin, Yukon Territory

HIGHLIGHTS

- Experimentation with traditional medicine and food plants that can grow in fuel breaks while converting the fuel break to a deciduous and more fire-resistant forest
- Maintenance of conversion from flammable coniferous forest to more fire-resistant deciduous forest through community wood harvesting.

CO-BENEFITS

Local collection and propagation of birch and herbaceous plant seeds, planting and transplanting of other local food and medicinal plants.

WHO WAS INVOLVED?

Teslin Tlingit Council (TTC) Lands Department, Government of Yukon (YG), Village of Teslin (VoT) set out to design the fuel break and plan for co-benefits.

WHAT WAS THE INTENTION?

- Protect Teslin from wildfire.
- Create easily accessible harvesting opportunities for firewood, medicinal plants, food, aesthetics, and promoting a conversion to less flammable deciduous forest.

WHAT WAS DONE?

The community of Teslin, Yukon Territory, was showing increased demand for fuelwood, sawlogs, and biomass for energy. In response, the Teslin Tlingit Council, Yukon Government, and the Teslin Renewable Resources Council developed a new Timber Harvest Plan, which is required to allow the commercial harvest of wood.

The management objectives in the timber harvest plan provide wood for the community, but also to manage the timber harvest to reduce wildland fire risk. Researchers from Yukon University are collaborating to determine what plant species the Teslin community would like to see growing in the wildfire fuel treatment and wood harvesting areas. They are also determining if local seed could be used to grow birch trees to help the forest convert to more deciduous and less flammable forest. They are planting other food and medicinal plants that are important to Teslin residents in the fuel treatment area to see if they will thrive.

To make the fuel break, they completed 100% canopy removal, given that there are other opportunities to examine areas cut to FireSmart standards. Timber harvest was done in a manner typical of how local woodcutters harvest Therefore, there were some scattered brush and burn 'scars' where the woodcutters burned the slash.

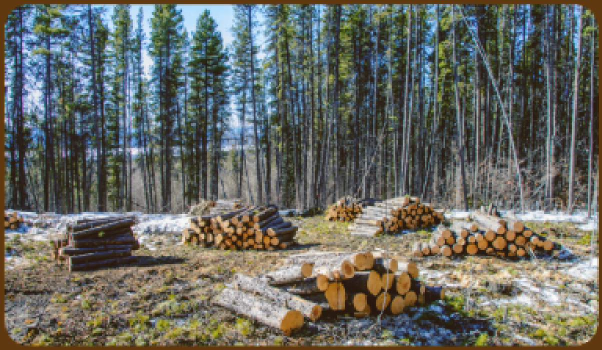

FUEL BREAK DETAILS

The area is a conifer-dominated hill that is North of the main town area. The area has a semi-continental boreal climate inland of coastal mountains and near a large lake, with cool summers and relatively mild winters. There is limited permafrost in the region. The timber harvest area is on a predominantly south- and east-facing slope. The soil is primarily shallow glacial till over fractured bedrock, with a thin cap of silt-loam loess soils and aa shallow organic layer (2-8 cm thickness). The forest canopy is dominated by lodgepole pine, mixed with white spruce, aspen, and birch. Green alder and high bush cranberry are common in the understory, with a mixture of lichen and feather moss on the forest floor.

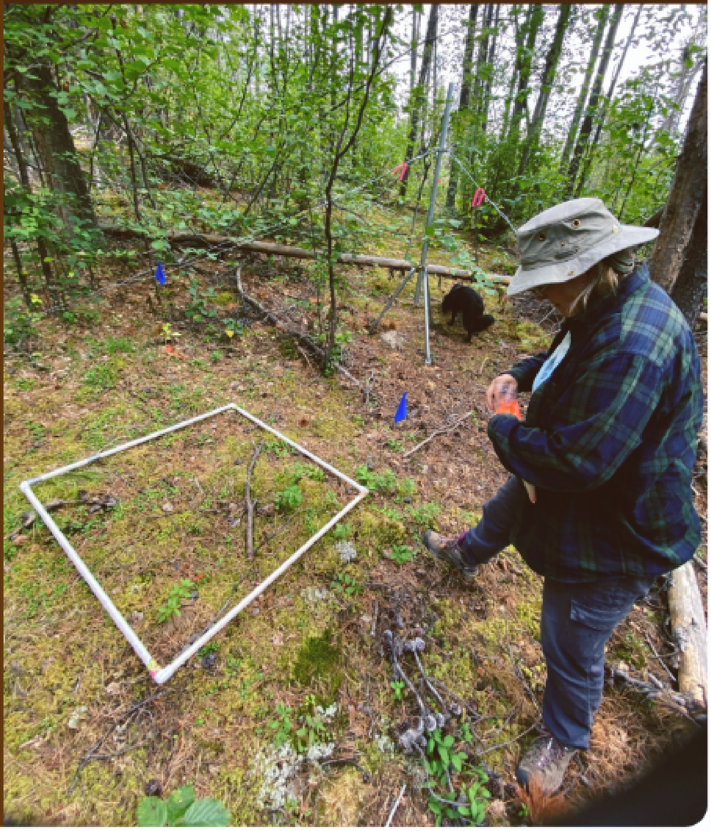

The "Fuel Breaks with Benefits" project acknowledges support from the National Center for Ecological Analysis and Synthesis (Morpho Initiative), US National Science Foundation (OPP 2332346), Bonanza Creek Long-Term Ecological Research Program, and University of Alaska Fairbanks.

UAF is an affirmative action/equal opportunity employer, educational institution and provider and prohibits illegal discrimination against any individual: www.alaska.edu/nondiscrimination.

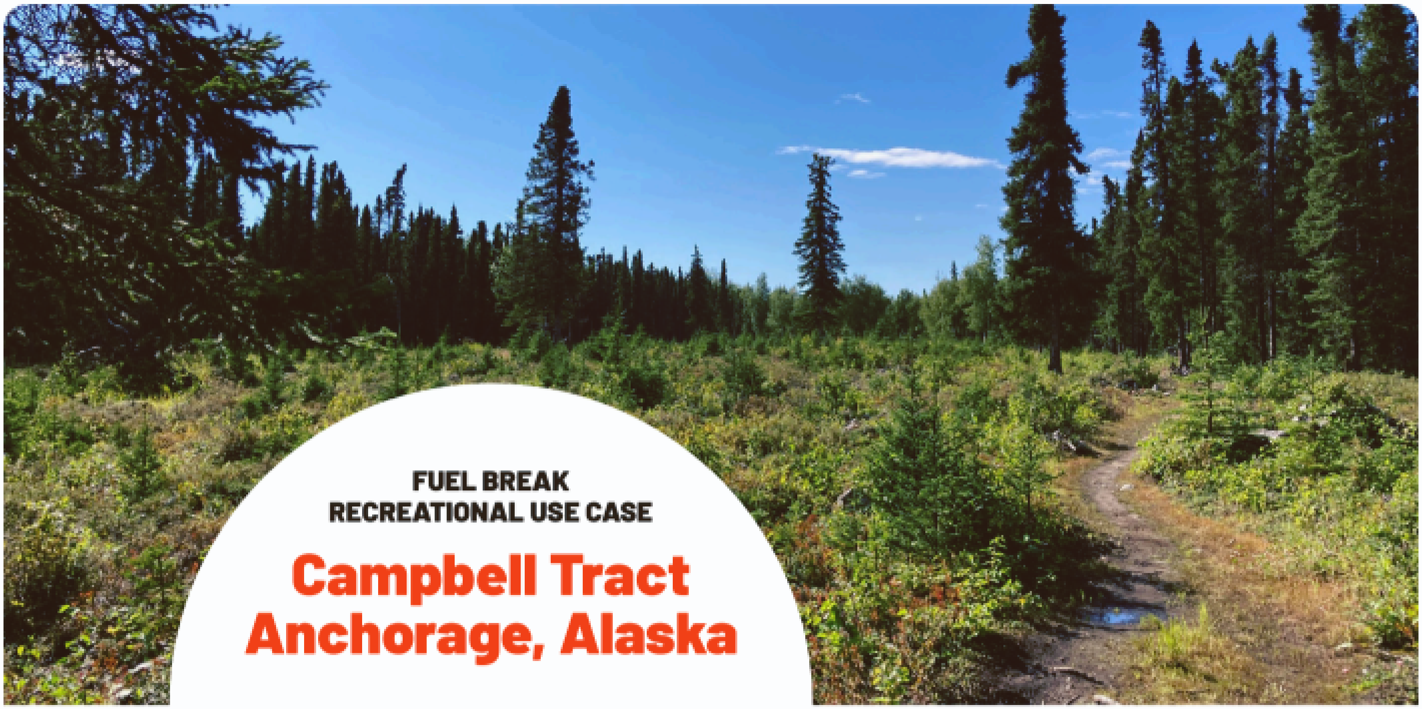

FUEL BREAK
RECREATIONAL USE CASE

Campbell Tract Anchorage, Alaska

WHAT WAS DONE

The Campbell Tract in Anchorage, Alaska was originally a military installation during World War II, and is now a public multi-use recreation area managed by the Bureau of Land Management. Because it is located at a critical wildland-urban interface (WUI) for wildfire, community groups and agencies in Anchorage created a fuel break in this city parkland in 2001 to prevent fire from moving from one part of town to another. The additional community benefits were not planned, but happened spontaneously and expanded the existing trails network, visibility in the forest, and educational opportunities. The fuel break is 33 acres and approximately 2 miles long.

HIGHLIGHTS

- The trail network is popular for walking, running, mountain biking, horseback riding, and wildlife viewing in the summer and snowshoeing, fat tire biking, skijoring, and cross country skiing in the winter.
- The Campbell Creek Science Center offers educational programs along the trail system and in the fuel break.
- Over 500,000 visitors used the trail network in 2022.
- The area was used in the ceremonial start of Iditarod sled dog race and one of Alaska's largest cross-country ski races.

WHO BENEFITS?

Multi-use activity in a wildland-urban interface: In 2020, the area had close to 400,000 visitors using the broader trail network! High public profile: used in the ceremonial start of Iditarod sled dog race and one of Alaska's largest cross-country ski races.

CO-BENEFITS

Recreation: Anchorage residents and tourists use the fuel break areas as an expansion of a non-motorized multi-use trail network.

Research and education: The fuel break has been used for research on how to manage fire, for vegetation monitoring plots, and for school field trips run by the science center.

HOW DID THEY DO IT?

In the **birch forest habitats**, they created a shaded fuel break by removing conifer trees.

In the **black spruce forest**, they performed initial thinning and pruning of trees, followed by removal of fallen trees and subsequent clearing. Now it is an open canopy fuel break.

Wetland habitats act as a natural fuel break connecting treated areas.

WHO WAS INVOLVED?

Planning: Municipal fire dept and BLM

Implementation: Municipal fire dept, BLM Alaska Fire Service, State Forestry

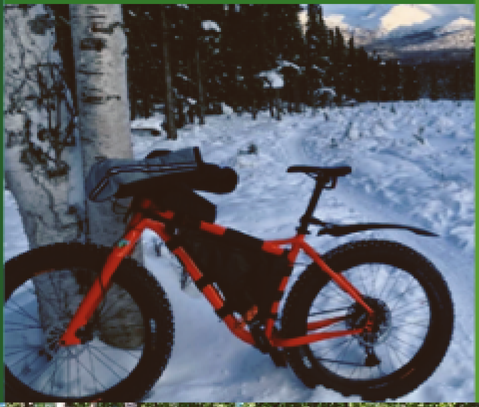

FUEL BREAK DETAILS

This linear fuel break (~200 ft or 60 m wide) is designed to limit spread of fire through parkland adjacent to city neighborhoods. The fuel treatments were done by hand crews.

BLM holds volunteer days with loppers to remove regenerating spruce as well as performs regular maintenance to remove fallen trees.

Left: The winter trails in the Campbell Tract provide increased opportunity for fat biking in close proximity to the city. PHOTO: BLM

Bottom: A shaded fuel treatment in the Campbell Tract provides areas for berry picking and hiking. PHOTO CREDIT: N. LOJEWSKI

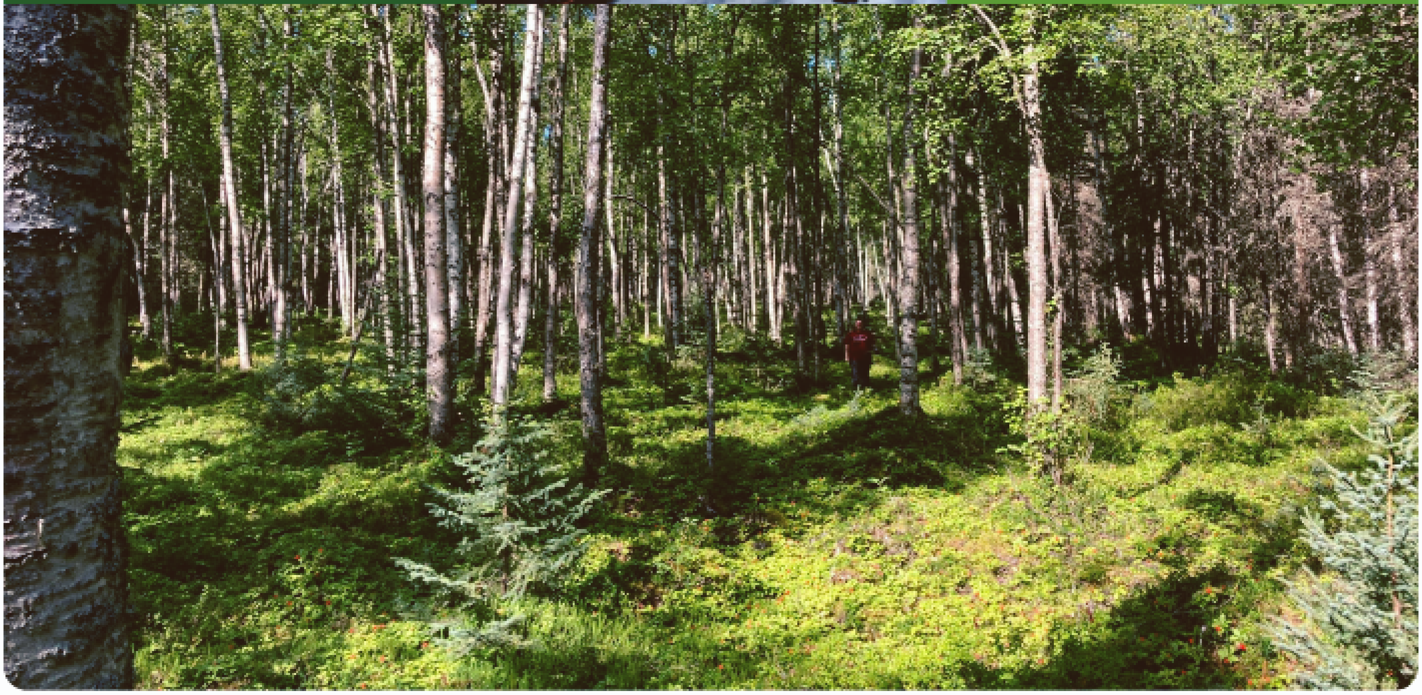

The "Fuel Breaks with Benefits" project acknowledges support from the National Center for Ecological Analysis and Synthesis (Morpho Initiative), US National Science Foundation (OPP 2332346), Bonanza Creek Long-Term Ecological Research Program, and University of Alaska Fairbanks.

UAF is an affirmative action/equal opportunity employer, educational institution and provider and prohibits illegal discrimination against any individual: www.alaska.edu/nondiscrimination.

Fuel Break Planning Roadmap Booklet

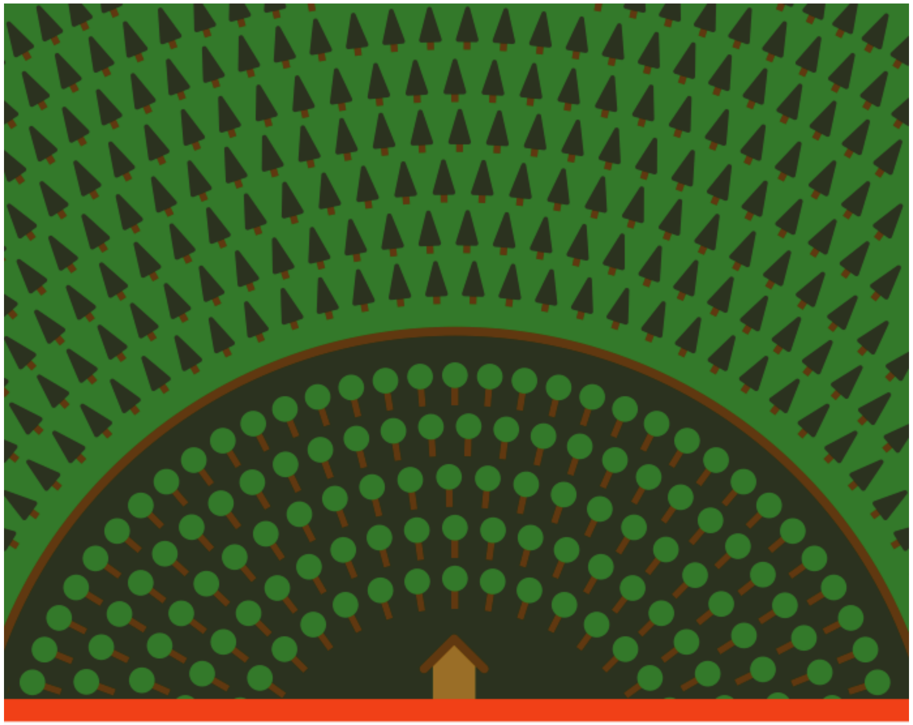

INTRODUCTION

This booklet supports the integration of community benefits into fuel break planning in boreal forests. It can be used alongside other tools like Community Wildfire Protection Plans and technical fuel break resources. Here we outline several broad steps to ensure the fuel break meets your community's needs.

Fuel breaks with benefits can enhance firefighters' ability to manage wildfires around communities and provide additional benefits to the community throughout the year. There are different fuel break options depending on your community's priorities and resources. Some require consistent maintenance and investment of time, resources, and people-power over many years. Other fuel break benefits require less investment or maintenance over time.

The "Fuel Breaks with Benefits" project acknowledges support from the National Center for Ecological Analysis and Synthesis (Morpho Initiative), US National Science Foundation (OPP 2332346), Bonanza Creek Long-Term Ecological Research Program, and University of Alaska Fairbanks.

UAF is an affirmative action/equal opportunity employer, educational institution and provider and prohibits illegal discrimination against any individual: www.alaska.edu/nondiscrimination.

Fuel Breaks with Benefits

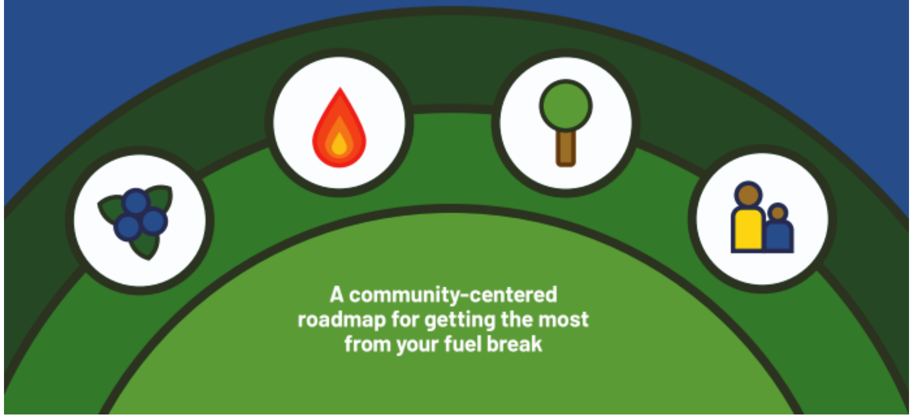

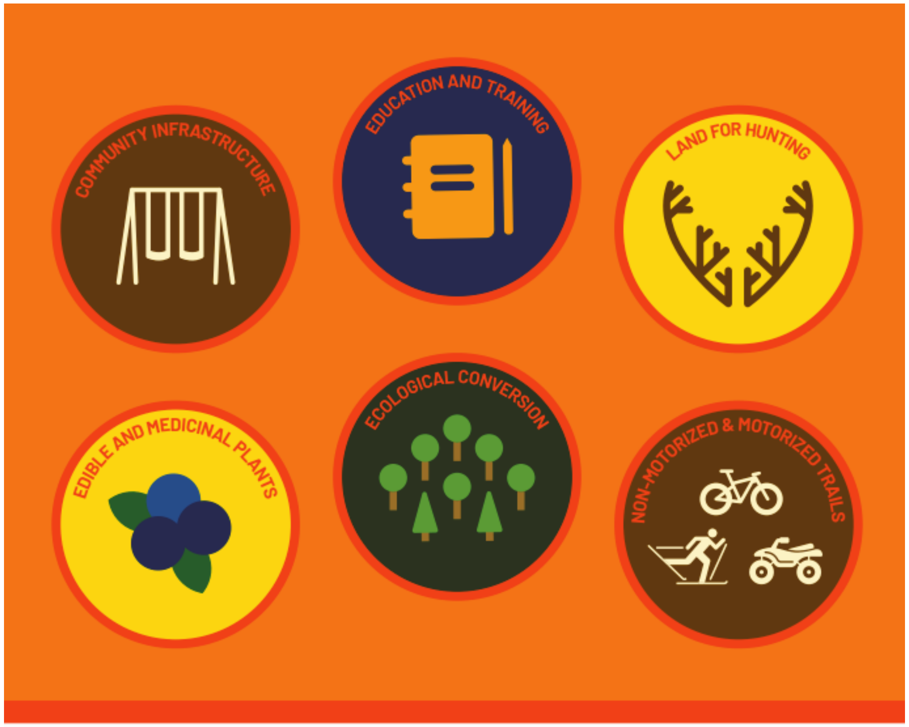

STEP 1
ENVISION

All fuel breaks with community benefits require an initial process that should, among other things:

- Identify what type of fuel break(s) you are creating based on your wildfire protection needs
- Collaboratively identify holistic community priorities
- Consider how the fuel break will be used for fire suppression
- Gather input about potential community benefits and discuss the feasibility of including them in the fuel break. Participatory mapping, roundtable discussions, community workshops, or other multi-stakeholder collaborative exercises are helpful here
- Assemble partnerships for implementation and maintenance
- Compare and communicate the risks of implementing a fuel break vs. not doing anything

In most situations, a Community Wildfire Protection Plan (CWPP) can meet these goals. In other situations alternative planning processes, including Firewise, may be more helpful or complementary.

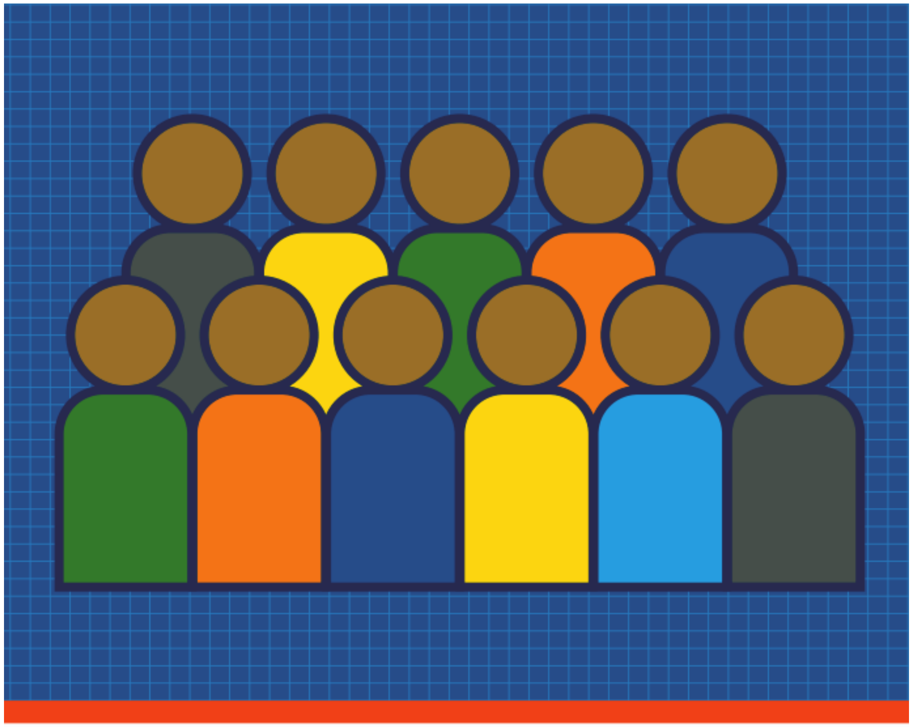

STEP 2
PLAN

To plan a fuel break with benefits, communities and individuals should:

- Create goals and objectives for the proposed project
- Recruit subject matter experts to inform your community's ideas, such as local and Traditional Knowledge holders, foresters, researchers, engineers, and fire practitioners
- Evaluate trade-offs, including costs, environmental constraints, fuel break effectiveness, cultural practices, permitting, community priorities, location, and methods of fuel break construction
- Realistically consider how the changes you are proposing will be used by the community to avoid unintended consequences
- Decide the who and how of implementation
- Engage local residents in planning, including discussions of the responsibilities of homeowners
- Apply for any necessary permits

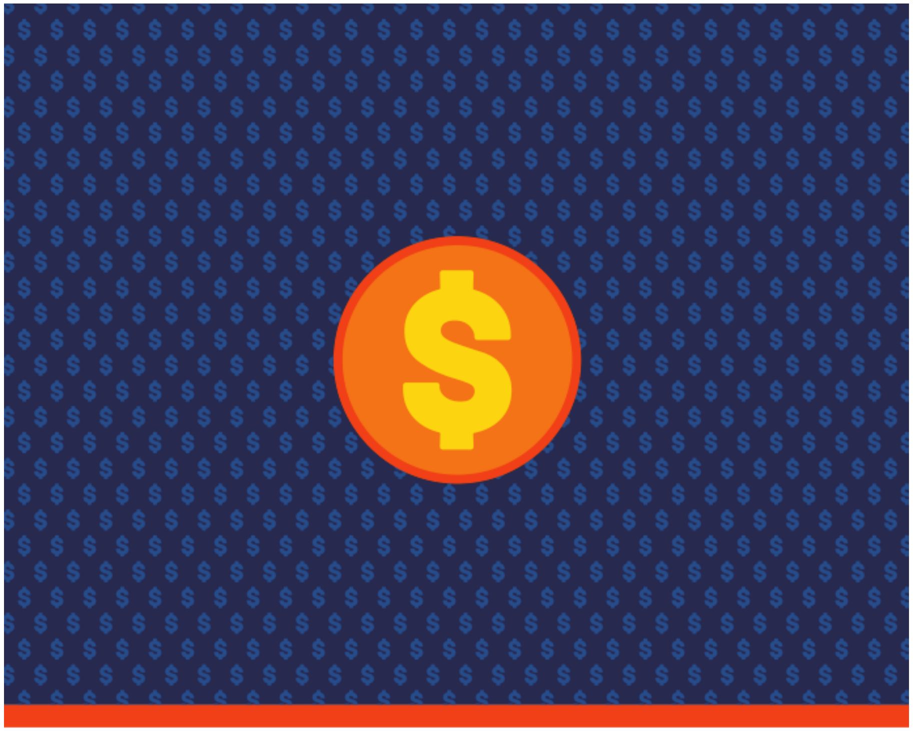

STEP 3

FUND

Multi-use fuel breaks can be expensive, but funding is available from a range of sources (including creative and unexpected ones) depending on:

- The goal of the fuel break
- Community benefits, including culture, health, recreation, food security, tourism, etc.
- Land ownership
- Potential for timber, firewood, biochar creation or other forest products
- Self-sufficiency or commercial opportunities

Consider funding from:

- Federal, territorial/state, regional or local government (consider different departments or agencies depending on the benefits you are interested in)
- Research partnerships
- Philanthropy or NGOs
- Industry

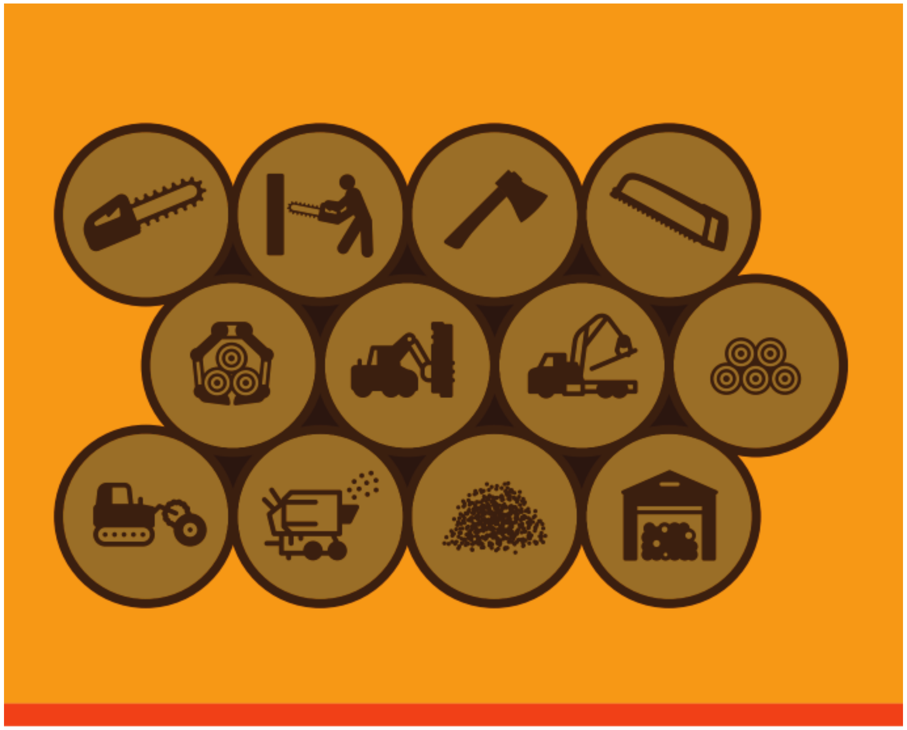

STEP 4

BUILD

Building a fuel break with benefits requires coordination with your community as well as various partners and experts, including foresters, Traditional Knowledge holders, researchers, engineers, and fire practitioners.

- Communicate timelines and potential disruptions to your community. The construction timeline of your fuel break will depend on season, weather, permitting, worker availability, methods of construction and type of fuel break
- Collaborate with partners to implement the shared vision

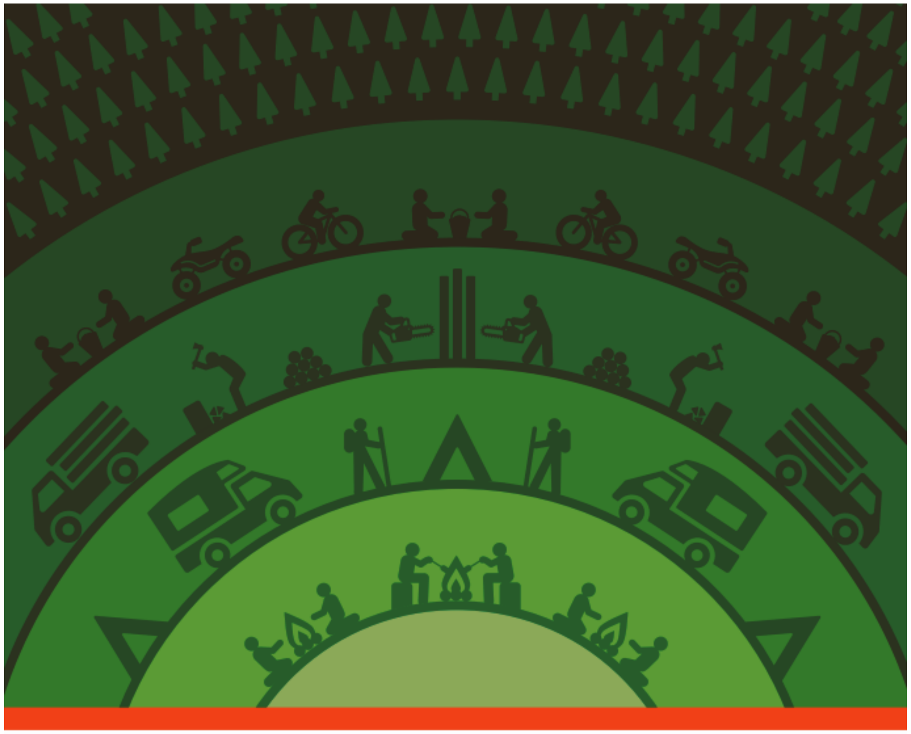

STEP 5

SUSTAIN

While initial construction demands particularly intense effort, regular and strategic maintenance will ensure the fuel break continues to benefit and protect your community long-term. The maintenance specifics will depend on your community's goals and will likely require flexibility and adaptability throughout the lifetime of the fuel break.

- Community education and participation can help to maintain and support the fuel break
- Consider site monitoring at regular intervals
- Invite people in your community to engage with the benefits that your fuel break has to offer:
  - recreation
  - prescribed and cultural fire
  - food security and harvesting
  - cultural activities
  - research

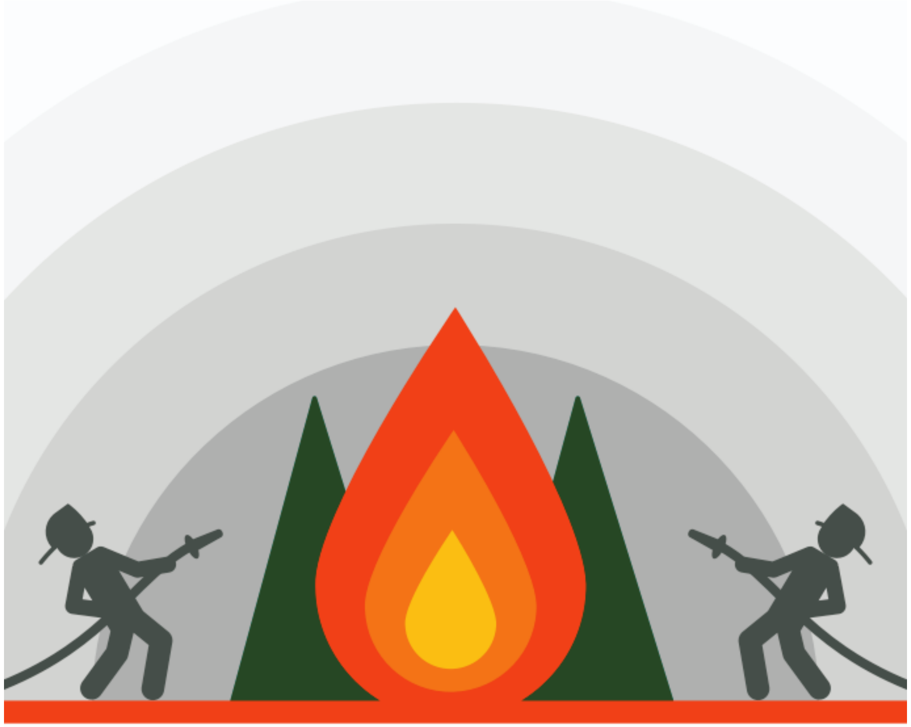

STEP 6

PROTECT

Fuel breaks change wildfire dynamics and can enhance the ability of fire protection crews to slow the progression of wildfires, allow time for evacuations and protect your community and infrastructure.

In the event of a wildfire, the fuel break can:

- Create space for the safe deployment of personnel, machinery and aircraft to respond to wildfires
- Support air tankers in effectively hitting targets
- Provide an anchor from which to action fires
- Create educational opportunities about fire evacuations and risk to homes in the wildland-urban interface
